# m^6^A readers ECT2/ECT3/ECT4 enhance mRNA stability through direct recruitment of the poly(A) binding proteins in *Arabidopsis*

**DOI:** 10.1186/s13059-023-02947-4

**Published:** 2023-04-30

**Authors:** Peizhe Song, Lianhuan Wei, Zixin Chen, Zhihe Cai, Qiang Lu, Chunling Wang, Enlin Tian, Guifang Jia

**Affiliations:** 1 Synthetic and Functional Biomolecules Center, Key Laboratory of Bioorganic Chemistry and Molecular Engineering of Ministry of Education, College of Chemistry and Molecular Engineering, Beijing National Laboratory for Molecular Sciences, Peking University, Beijing, 100871 China; 2 Peking-Tsinghua Center for Life Sciences, Beijing, 100871 China

**Keywords:** *N*^6^-Methyladenosine (m^6^A), m^6^A readers ECT2/ECT3/ECT4, Stability, PAB proteins

## Abstract

**Background:**

RNA *N*^6^-methyladenosine (m^6^A) modification is critical for plant growth and crop yield. m^6^A reader proteins can recognize m^6^A modifications to facilitate the functions of m^6^A in gene regulation. ECT2, ECT3, and ECT4 are m^6^A readers that are known to redundantly regulate trichome branching and leaf growth, but their molecular functions remain unclear.

**Results:**

Here, we show that ECT2, ECT3, and ECT4 directly interact with each other in the cytoplasm and perform genetically redundant functions in abscisic acid (ABA) response regulation during seed germination and post-germination growth. We reveal that ECT2/ECT3/ECT4 promote the stabilization of their targeted m^6^A-modified mRNAs, but have no function in alternative polyadenylation and translation. We find that ECT2 directly interacts with the poly(A) binding proteins, PAB2 and PAB4, and maintains the stabilization of m^6^A-modified mRNAs. Disruption of *ECT2/ECT3/ECT4* destabilizes mRNAs of ABA signaling-related genes, thereby promoting the accumulation of ABI5 and leading to ABA hypersensitivity.

**Conclusion:**

Our study reveals a unified functional model of m^6^A mediated by m^6^A readers in plants. In this model, ECT2/ECT3/ECT4 promote stabilization of their target mRNAs in the cytoplasm.

**Supplementary Information:**

The online version contains supplementary material available at 10.1186/s13059-023-02947-4.

## Background

Chemical modifications in RNA can regulate gene expression, critically affecting biological functions. *N*^6^-methyladenosine (m^6^A), the most abundant internal modification in eukaryotic mRNA, can be dynamically deposited, removed, and read by three types of proteins to modulate transcriptional and post-transcriptional gene expression [1–7]. In *Arabidopsis thaliana*, the m^6^A methyltransferase complex, consisting of mRNA adenosine methylase (MTA), methyltransferase B (MTB), FKBP12 interacting protein 37 (FIP37), VIRILIZER, and HAKAI, confers specificity for the majority of m^6^A depositions [8–11]. ALKBH9B and ALKBH10B are responsible for the m^6^A demethylation [12, 13]. The accumulating evidences suggest that m^6^A modification has fundamental regulatory roles in various plant biological processes. In *Arabidopsis*, m^6^A has been shown to modulate shoot stem cell proliferation, trichome branching, floral transition, abscisic acid (ABA) response, salt stress, cold stress, photosynthesis, and nitrate signaling [10, 14–23]. Additionally, m^6^A plays major regulatory roles in rice sporogenesis and grain yield, as well as in strawberry fruit ripening [24–26].

Despite the well-documented biological functions of m^6^A in crucial plant processes, the molecular mechanism underlying its regulatory roles remains poorly understood. Previous studies conducted in mammalian cells have suggested that the precise regulation of RNA metabolism by m^6^A is mainly achieved through mRNA recognition by YT521-B homology (YTH) domain-containing reader proteins [5, 6, 27]. Thirteen YTH family proteins have been identified in *Arabidopsis* through homology analysi*s*, namely Evolutionarily conserved C-terminal region (ECT)1–11, AT4G11970, and the longer isoform of Cleavage and polyadenylation specificity factor 30 (CPSF30-L) [15]. Currently, only ECT2, ECT3, ECT4, and CPSF30-L have been characterized as m^6^A reader proteins [14–18]. CPSF30-L is a nuclear m^6^A reader protein that regulates alternative polyadenylation (APA) of pre-mRNA in liquid-like nuclear bodies, where CPSF30-L recognizes the m^6^A-modified far upstream elements (FUE) polyadenylation signal to control poly(A) site choice [18]. ECT2 and ECT3 have redundant effects on trichome branching, while ECT2, ECT3, and ECT4 redundantly function in leaf growth and organogenesis [16, 28]. ECT2 regulates trichome branching by promoting trichome morphology-related mRNA stability [14]. ECT2 and ECT3 regulate the targeted mRNA abundance and their binding targets are largely overlapped [29]. However, many questions remain unanswered regarding the molecular functions of ECT2, ECT3, and ECT4, such as how ECT2 and ECT3 achieve largely overlapping targets in spatial terms, whether and how they stabilize m^6^A-modified mRNA to regulate mRNA abundance, and whether certain m^6^A sites are recognized by two different types of m^6^A readers, resulting in distinctive regulatory functions in RNA processing.

In this study, we demonstrate that the cytoplasmic m^6^A reader proteins, ECT2, ECT3, and ECT4, directly interact with each other to enhance the m^6^A-binding ability. They function redundantly in regulating ABA response during seed germination and post-germination growth. The ECT2/ECT3/ECT4 complex was determined to have no role in APA regulation or translation efficiency. By combining mRNA stability profiling and formaldehyde cross-linking and immunoprecipitation (FA-CLIP) data analysis, we revealed that ECT2/ECT3/ECT4 cooperatively promote stability of bound m^6^A-modified mRNAs, thereby affecting gene expression. We identified the mRNA stabilizers poly(A) binding protein 2 (PAB2) and PAB4 as ECT2 binding proteins that stabilize targeted m^6^A-modified mRNAs through direct recruitment. Deficiency in *ECT2/ECT3/ECT4* accelerated mRNA degradation of four ABA signaling-related genes and led to ABA hypersensitivity. Our integrated study revealed a novel model in which multiple m^6^A readers redundantly regulate m^6^A-mediated mRNA stabilization in plants.

## Results

### ECT2, ECT3, and ECT4 directly interact with each other and enhance the m^6^A-binding function

**Fig. 1:**
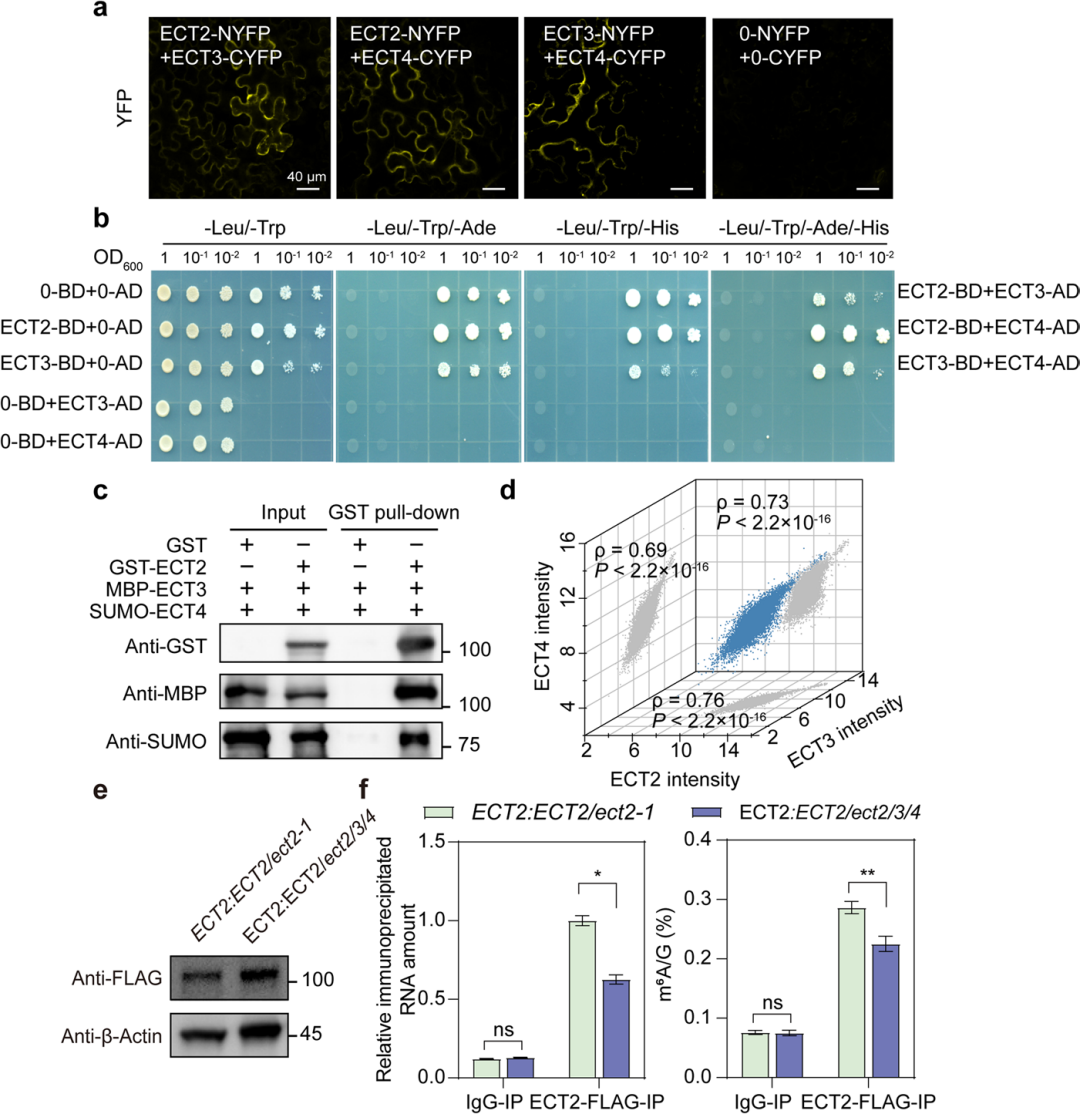
Direct protein–protein interaction among ECT2, ECT3, and ECT4. **a** BiFC assay showing the physical associations among ECT2, ECT3, and ECT4 in *Nicotiana benthamiana* leaf cells. The association of paired proteins is indicated by YFP fluorescence in the cytoplasm. Scale bars = 40 μm. **b** Y2H assay showing the physical associations among ECT2, ECT3, and ECT4 in yeast cells. The full-length coding sequences of ECT2, ECT3, and ECT4 were fused with either the GAL4-AD or BD domain as indicated. YSD-Leu-Trp-Ade-His, selective medium without tryptophan, leucine, histidine, or adenine; YSD-Leu-Trp, medium without tryptophan or leucine (growth control). **c** Pull-down assay showing the interaction among GST-ECT2, MBP-ECT3, and SUMO-ECT4 in vitro. Purified MBP-ECT3 and SUMO-ECT4 proteins were incubated with GST-ECT2 or GST alone, and pull-down assays were performed using GST magnetic beads, followed by immunoblot analysis with anti-GST, anti-MBP, and anti-SUMO antibodies. **d** Correlation analysis of mRNA expression levels in *Arabidopsis* among ECT2, ECT3, and ECT4 in the ATTED-II database (*n* > 10,000 samples; *ρ*, Spearman’s correlation coefficient). *P*-values were calculated with Pearson’s correlation analysis. **e **The protein level of ECT2–FLAG in indicated samples, as determined by western blot. β-Actin protein was used as the loading control. **f** In vivo FA-RIP assay showing that ECT2-IP RNA amount and m^6^A level is enriched in *ECT2:ECT2/ect2-1* compared with *ECT2:ECT2/ect2/3/4* plant. IgG-IP was used as control. Data are presented as means ± SE, *n* = 3 biological replicates × 2 technical replicates. *P < 0.05, **P < 0.01 (two-sided *t-*test)

Previous studies have demonstrated that m^6^A reader proteins ECT2 and ECT3 bind largely overlapping targeted sites and ECT2/ECT3/ECT4 participate redundantly in certain plant developmental processes, including plant developmental timing, morphogenesis, and plant organogenesis [16, 28, 29]. To investigate the spatial aspect of their overlapping targets and redundant regulation, we first utilized the bimolecular fluorescence complementation (BiFC) system to co-express ECT2, ECT3, and ECT4 in pairs with split yellow fluorescent protein (YFP) in *N. benthamiana* leaves. All three protein pairs, ECT2-nYFP + ECT3-cYFP, ECT2-nYFP + ECT4-cYFP, and ECT3-nYFP + ECT4-cYFP, exhibited strong reconstituted YFP signal in the cytoplasm. In contrast, no fluorescence signal was observed with empty vector co-expression and other negative controls (Fig. 1a; Additional file 1: Fig. S1). To follow up on these results, we performed yeast two-hybrid (Y2H) assays to examine pairwise interactions among the full-length ECT2, ECT3, and ECT4 proteins. Yeast strains co-transformed with ECT2-BD + ECT3-AD, ECT2-BD + ECT4-AD, and ECT3-BD + ECT4-AD successfully grew on selective medium at all dilutions (Fig. 1b), demonstrating the occurrence of protein–protein interactions.

Since both the BiFC and Y2H assays confirmed that ECT2, ECT3, and ECT4 directly interact with each other, we asked whether ECT2, ECT3, and ECT4 could form a complex via pairwise interaction. To test this hypothesis, we performed an in vitro glutathione S-transferase (GST) pull-down assays with purified recombinant proteins from *Escherichia coli* to examine whether ECT2 could physically interact with ECT3 and ECT4. We found that maltose-binding protein (MBP)-tagged ECT3 and (small ubiquitin-like modifier) SUMO-tagged ECT4 interacted with GST-tagged ECT2, but not with GST alone (Fig. 1c), suggesting that ECT2, ECT3, and ECT4 constituted a complex through direct protein–protein interactions in vitro. Additionally, the mRNA expression level landscape of *ECT2*, *ECT3*, and *ECT4* was strongly correlated in all three pairwise comparisons (Spearman’s *ρ* values between 0.69 and 0.76; Fig. 1d), indicating their largely undifferentiated functions in various plant biological and developmental processes.

To investigate the regulatory role of ECT2, ECT3, and ECT4 interaction in m^6^A-modified RNAs, we assessed whether the ECT2-ECT3-ECT4 interaction affects the m^6^A-binding activity of ECT2 by conducting a formaldehyde cross-linking and RNA immunoprecipitation (FA-RIP) assay using the generated ECT2 complementary transgenic plants in *ect2-1* and *ect2/3/4* background, respectively (*ECT2:ECT2/ect2-1* and *ECT2:ECT2/ect2/3/4*). Both *ECT2:ECT2/ect2-1* and *ECT2:ECT2/ect2/3/4* plants expressed FLAG-tagged ECT2 proteins at similar levels (Fig. 1e). Our findings demonstrated a significant decrease in the amount of immunoprecipitated RNA and m^6^A level in *ECT2:ECT2/ect2/3/4* compared to *ECT2:ECT2/ect2-1* (Fig. 1f), indicating that ECT2, in collaboration with ECT3 and ECT4, can bind more m^6^A-modified RNA than ECT2 alone. These observations established that ECT2/ECT3/ECT4 can form a complex through direct protein–protein interactions, thereby enhancing m^6^A-binding capability and conferring fine regulation on their target RNAs.

### ECT2/ECT3/ECT4 are required for seed germination and post-germination development under ABA treatment

Although the deficiency of *ECT2/ECT3/ECT4* has been shown to delay development in early growth stages [16, 28], it is not yet known whether they play a role in abiotic stress responses. To investigate the biological functions of ECT2/ECT3/ECT4, we generated homozygous T-DNA insertion double mutants *ect2-1/ect4-1* (referred to as *ect2/4*) and *ect3-2/ect4-1* (*ect3/4*), as well as a triple mutant, *ect2-1/ect3-2/ect4-1* (*ect2/3/4*), by crossing the *ect2-1* (SALK_002225) mutant with the *ect3-2* (GABIseq_487H12) and *ect4-1* (SALK_151516) mutants (Additional file 1: Fig. S2a). Reverse transcription quantitative PCR (RT-qPCR) and detailed phenotypic analysis confirmed knockout of the target genes among *ect2/4*, *ect3/4*, and *ect2/3/4* mutants and the *ect2/3/4* mutant exhibited defective leaf morphology under normal growth conditions (Additional file 1: Fig. S2b, c), consistent with a previous report [16].

**Fig. 2:**
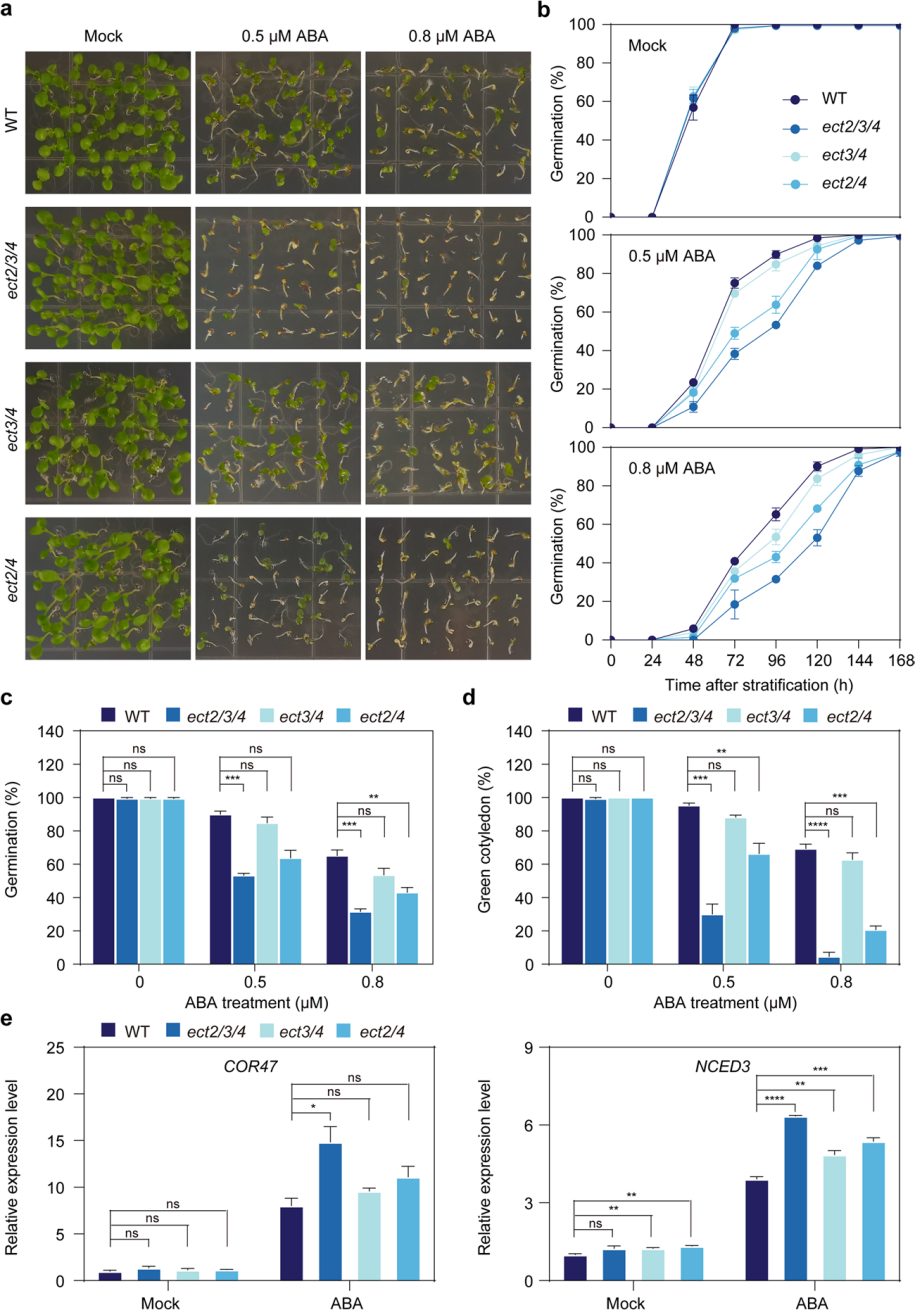
ECT2/ECT3/ECT4 are required for seed germination and post-germination development under ABA treatment. **a** Phenotypic analysis of the ABA response in WT, *ect2/3/4*, *ect3/4*, and *ect2/4* seeds grown on 1/2 MS-medium supplemented with 0 (Mock), 0.5, or 0.8 μM ABA under long-day conditions. Representative photographs were taken 8 days after cold stratification. **b **Statistical analysis of germination rates in WT, *ect2/3/4, ect3/4*, and *ect2/4* plants under ABA treatment. Radicle emergence was used as the morphological marker for germination. At least 40 seeds per genotype were measured in each replicate. Biological triplicates were averaged. Data are presented as the mean ± SE. **c–d** Statistical analysis of germination rates 4 days after imbibition (**c**) and of cotyledon greening rates 8 days after imbibition (**d**) in WT, *ect2/3/4, ect3/4*, and *ect2/4* plants under ABA treatment. Data are presented as the mean ± SE; *n* = 3 biological replicates. **P < 0.001, ***P < 0.001, ****P < 0.0001 (two-sided *t-*test). **e** Relative mRNA levels of *COR47* and *NCED3* in 7-day-old WT, *ect2/3/4, ect3/4*, and *ect2/4* seedlings under Mock and ABA treatment. *TUB8* was used as the internal control gene. Data are presented as the mean ± SE; *n* = 3 biological replicates × 2 technical replicates. *P < 0.05, **P < 0.01, ***P < 0.001, ****P < 0.0001 (two-sided *t-*test)

To investigate the roles of ECT2, ECT3, and ECT4 in abiotic stress responses, we initially assessed their ABA sensitivity by measuring the germination rate of single mutant seeds (*ect2-1*, *ect3-2*, and *ect4-1*). No obvious differences in germination rates were observed between the wild-type (WT) and mutant seeds under normal conditions (Mock) or with varying ABA concentrations (Additional file 1: Fig. S3a-c). As ABA is known to inhibit cotyledon greening more strongly than germination [30], we also evaluated the cotyledon greening rates and found *ect2-1* exhibited a significant reduction in greening compared to WT in the presence of ABA. The *ect3-2* and *ect4-1* mutants showed only a slight reduction (not statistically significant) in cotyledon greening rates upon ABA treatment (Additional file 1: Fig. S3d). We next examined ABA sensitivity in double and triple mutant seeds (*ect2/4*, *ect3/4*, and *ect2/3/4*) and found that in the presence of different concentrations of ABA, all mutant seeds all exhibited enhanced ABA sensitivity compared to WT in a manner that demonstrated genetic redundancy; ABA hypersensitivity in *ect3/4* was weaker than that of *ect2/4*, and *ect2/3/4* seeds exhibited stronger ABA hypersensitivity than either *ect2/4* or *ect3/4* mutants (Fig. 2a–d). These results demonstrated that ECT2/ECT3/ECT4 redundantly and negatively regulate ABA signaling during seed germination and post-germination growth.

In addition, we found that expression levels of some ABA-responsive genes were modulated by ECT2/ECT3/ECT4 activity. In the presence of exogenous ABA, the *ect2/3/4*, *ect3/4*, and *ect2/4* mutants showed up-regulation of ABA-responsive genes such as *COLD-REGULATED 47* (*COR47*) and *NINE-CIS-EPOXYCAROTENOID DIOXYGENASE 3* (*NCED3*). Among the three double/triple mutants, these stress-responsive genes were most significantly up-regulated in *ect2/3/4* compared to the WT (Fig. 2e). These results confirmed that ECT2/ECT3/ECT4 redundantly function in the expression of ABA-related transcripts during seed germination and post-germination growth.

Considering the observation that the absence of ECT2 protein exhibited the reduced cotyledon greening rate, and the presence of ECT2 protein could largely rescue the ABA sensitivity phenotype, we next assessed whether ECT2 predominately depends on its m^6^A-binding ability to function in the ABA response. We generated *ECT2:ECT2/ect2/3/4* and *ECT2:ECT2m/ect2/3/4* transgenic plants, which expressed the coding sequence of ECT2 and the m^6^A-binding abolished ECT2m with double mutations (W521A/W534A) [14] in the *ect2/3/4* mutant background. The germination and cotyledon greening rates of the transgenic seeds were indistinguishable from WT seeds under Mock treatment (Additional file 1: Fig. S4a, b). However, in the presence of ABA, *ECT2:ECT2/ect2/3/4* but not *ECT2:ECT2m/ect2/3/4* can partially rescue the ABA hypersensitivity in the *ect2/3/4* mutant (Additional file 1: Fig. S4). These results suggest that the m^6^A binding function plays a core regulatory role in ECT2/ECT3/ECT4-mediated ABA response.

### ECT2/ECT3/ECT4 promote mRNA stabilization

**Fig. 3:**
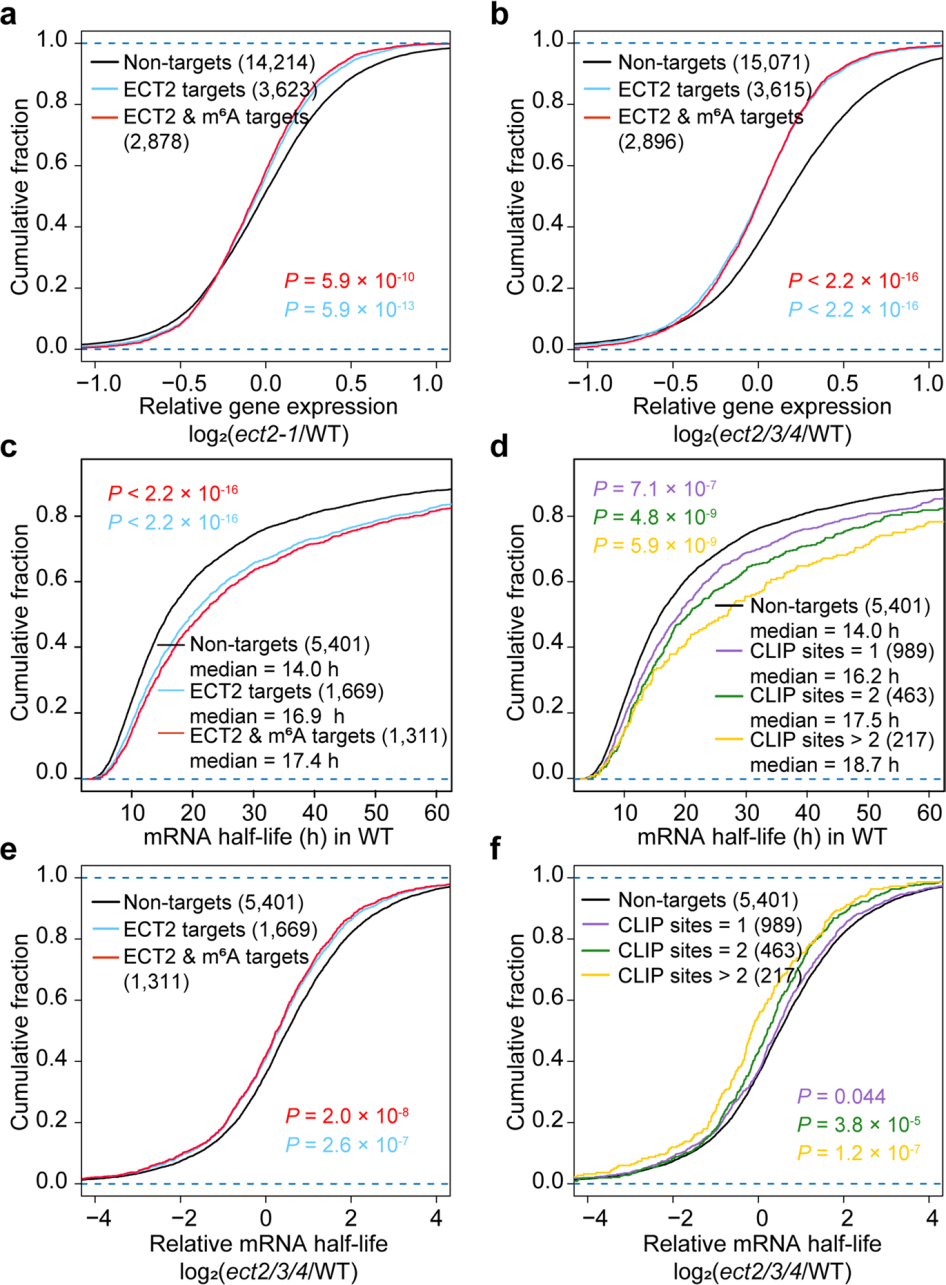
ECT2/ECT3/ECT4 promote stabilization of target m^6^A-modified mRNA. **a**–**b** Cumulative distribution of relative mRNA expression in *ect2-1* compared to WT (**a**) and *ect2/3/4* compared to WT (**b**) for Non-targets (black), ECT2 targets (blue), and ECT2 & m^6^A targets (red). **c** Cumulative distribution of mRNA half-life in WT for Non-targets (black), ECT2 targets (blue), and ECT2 & m^6^A targets (red). **d** Cumulative distribution of mRNA half-life in WT, analyzed based on the number of ECT2 target sites in each transcript. **e** Cumulative distribution of relative mRNA half-life log_2_ fold changes between *ect2/3/4* and WT for Non-targets (black), ECT2 targets (blue), and ECT2 & m^6^A targets (red). **f** Cumulative distribution of mRNA half-life log_2_ fold changes between *ect2/3/4* and WT, analyzed based on the number of ECT2 target sites in each transcript. *P*-values were calculated using two-sided Mann–Whitney *U* test

Our previous studies have shown that ECT2 promotes mRNA stability in the cytoplasm [14] (Fig. 3a). However, it is unclear whether ECT2/ECT3/ECT4 cooperatively facilitate mRNA stabilization. To investigate this, we performed mRNA sequencing (mRNA-seq) in WT and *ect2/3/4* seedlings. Correlation analysis between two biological replicates for each genotype confirmed that the replicability of the mRNA-seq (Additional file 1: Fig. S5). Transcripts with per million mapped fragments (FPKM) < 1 were excluded. Considering that more than 94% of ECT3 targets were overlapped with ECT2 targets [29], and the predominant phenotype-related regulatory role of ECT2 in ECT2/ECT3/ECT4 (Fig. 2a), we chose ECT2 targets from previous FA-CLIP-seq [14] that could largely cover the targets of ECT2, ECT3, and ECT4 to analyze the datasets. We divided the genes into three groups: ECT2 targets, ECT2 & m^6^A targets (ECT2-binding genes with m^6^A modification), and Non-targets (ECT2 unbound genes). Our results showed that the *ect2/3/4* mutant had significantly lower accumulation of ECT2 targets and of ECT2 & m^6^A targets compared to non-targets (Fig. 3b). This trend of reduced transcript accumulation was stronger in the *ect2/3/4* than in the *ect2-1* mutant (Fig. 3a, b), consistent with the functional redundancy of ECT2/ECT3/ECT4.

We subsequently performed mRNA stability profiling by calibrating External RNA Controls Consortium (ERCC) spike-in controls in equal amounts of total RNA from 7-day-old WT and *ect2/3/4* mutant plants to investigate the functional role of ECT2/ECT3/ECT4 in mRNA stabilization. Plants were collected over a series of time points after transcription inhibition with actinomycin D. Analysis results revealed that both ECT2 targets and ECT2 & m^6^A targets tended to have longer mRNA half-lives than Non-targets in WT (Fig. 3c; Additional file 2: Table S1). We also examined whether there was a correlation between mRNA stability and the number of ECT2-binding sites by dividing the ECT2 targets into three groups based on the number of ECT2-binding sites they had. A positive association was observed between the number of ECT2-binding sites and mRNA target stability, with targets having more than two ECT2-binding sites showing increased stability compared to targets with only one or two binding sites (Fig. 3d). Moreover, we found that compared with WT, the mRNA half-lives of ECT2 and ECT2 & m^6^A targets were significantly shortened in the *ect2/3/4* mutants relative to Non-targets (Fig. 3e). This analysis also revealed that mRNA stabilization was again associated with the number of ECT2-binding sites (Fig. 3f; Additional file 2: Table S1). We further examined individual genes and found that some ABA-related transcripts bound by ECT2 and modified with m^6^A were rapidly degraded in *ect2/3/4* mutant (Additional file 1: Fig. S6), suggesting that ECT2/ECT3/ECT4 function in ABA response via enhancing mRNA stabilization.

Additionally, we analyzed the previously identified ECT2/ECT3 common targets [29] with our mRNA stability profiling data. Consistently, both ECT2/ECT3 common targets had longer mRNA half-lives than their Non-targets in WT (Additional file 1: Fig. S7a). Disruption of ECT2/ECT3/ECT4 reduced mRNA half-lives for ECT2/ECT3 common targets compared with Non-targets (Additional file 1: Fig. S7b). These findings provide compelling evidence that the m^6^A reader proteins ECT2, ECT3, and ECT4 act in concert to promote mRNA stabilization in *Arabidopsis*, highlighting a previously unrecognized regulatory mechanism for m^6^A modification in plant RNA metabolism.

### ECT2/ECT3/ECT4 have no effects on alternative polyadenylation and translation

Our previous findings suggested that ECT2 may be involved in mRNA 3′ end processing due to its binding around the UGUA region [14]. However, a recent study using NanoPARE analysis revealed that ECT2/ECT3/ECT4 do not play a direct role in alternative polyadenylation [29]. We further investigate whether ECT2/ECT3/ECT4 affect alternative polyadenylation (APA). The subcellular localization of m^6^A readers is known to influence their regulatory roles in RNA processing and metabolic processes. Thus, *ect2-1* mutant plants that expressed a transgene for ECT2 fused to green fluorescence protein (eGFP) were utilized to acquired high-resolution images of ECT2-eGFP in 7-day-old root tips. The results confirmed the cytoplasmic localization of ECT2 (Additional file 1: Fig. S8), suggesting that ECT2 would have negligible effects on RNA processing in the nucleus. To investigate further, we sequenced polyadenylation (poly(A)) sites using the A-seq2 method [31] in WT and *ect2/3/4* plant samples. Microheterogeneity at the cleavage and poly(A) site often produces clusters of related poly(A) sites [32]. We therefore consolidated all sites ending within 25 nucleotides of one another into a single poly(A) cluster (PAC) for further analysis and identified over 19,000 high-confidence PACs per sample (tags per million (TPM) ≥ 3) after mapping ~ 10 million reads per sample to the *Arabidopsis* genome. More than 80% of PACs aligned to the terminal exons and 3′ untranslated regions (3′ UTRs) of protein-coding genes (Additional file 1: Fig. S9a) and correlation analysis between biological replicates confirmed the reproducibility of the poly(A) site profiling results (Additional file 1: Fig. S9b).

We identified a total of 128 genes with high-confidence PAC shifts (*P*-value < 0.05, Fisher’s exact test) in the *ect2/3/4* mutant, which only accounted for 1.34% of all genes with a detected PAC (Additional file 1: Fig. S10a, b). To examine whether ECT2/ECT3/ECT4 were associated with the 128 PAC-shifted genes, we calculated the percentage of PAC-shifted genes in the three groups described above: ECT2 targets, ECT2 & m^6^A targets, and Non-targets. The results showed that the PAC shifting rate was comparable between ECT2 targets, ECT2 & m^6^A targets, and Non-targets (Additional file 1: Fig. S10a, c). Because PAC shifting would affect 3′ UTR length, we compared 3′ UTR length between *ect2/3/4* mutant and WT plants using the same mRNA groupings. The results showed that there were no significant differences in 3′ UTR length in ECT2 targets and ECT2 & m^6^A targets compared to Non-targets (two-sided *t*-test; Additional file 1: Fig. S10d). Thus, we conclude that ECT2/ECT3/ECT4 are not responsible for the APA processing of m^6^A-modified genes.

Considering the cytoplasmic localization of ECT2, we evaluated their impact on translation efficiency in WT seedlings where mRNAs were recognized by ECT2 or ECT2/ECT3/ECT4 and in *ect2-1* or *ect2/3/4* seedlings where mRNAs were not recognized by these m^6^A reader proteins. Ribosome profiling (ribo-seq) was performed to measure the translation efficiency (ribosome-bound fragments/mRNA input) of targeted mRNAs (Additional file 3: Table S2). Ribosome-bound fragments were generated with high reproducibility between biological replicates through nuclease digestion of polysomes into monosomes (Additional file 1: Fig. S11a-d). Our analysis showed no significant differences in translation efficiency between ECT2 or ECT2 & m^6^A targets and Non-targets in *ect2-1*, *ect2/3/4*, or WT plants (Additional file 1: Fig. S11e, f). This suggests that ECT2/ECT3/ECT4 have no function in protein translation.

### The PrLDs of ECT2 directly interacts with PAB2 and PAB4

**Fig. 4:**
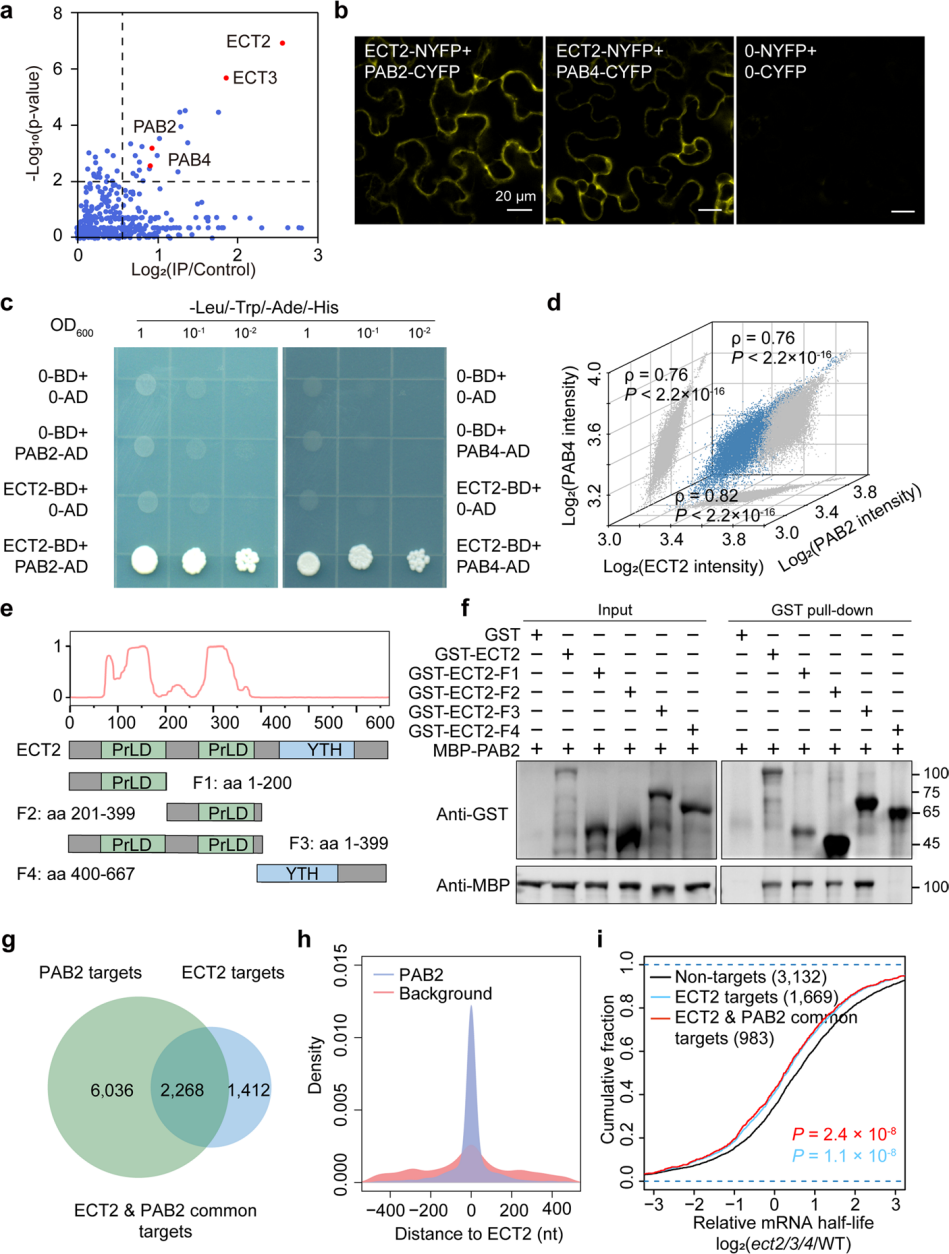
ECT2/ECT3/ECT4 promote mRNA stability by recruiting PAB proteins. **a** Scatterplot showing the proteins bound to endogenous *Arabidopsis* ECT2 after RNase T1 treatment. The plot is based on the enrichment level (IP/control) and *P-*value. **b **BiFC assay showing the physical interaction between ECT2 and PAB2/PAB4 in *Nicotiana benthamiana* leaf cells. Scale bars = 20 μm. **c** Y2H assay showing the interaction of ECT2 with PAB2 and with PAB4 in yeast cells. **d** Correlation analysis of mRNA expression levels in *Arabidopsis* among ECT2 and PAB2, and PAB4 in the ATTED-II database (*n* > 10,000 samples; *ρ*, Spearman’s correlation coefficient). *P*-values were calculated with Pearson’s correlation analysis. **e** Top: predictions of PrLDs made by the “prion-like amino acid composition” (PLAAC; http://plaac.wi.mit.edu/); bottom: schematic diagram of ECT2 and its fragments. **f **Pull-down assay showing a direct interaction between PAB2 and PrLD domain of ECT2 in vitro. Purified MBP-PAB2 was incubated with GST-ECT2 fragments or GST alone, and pull-down assays were performed using GST magnetic beads, followed by immunoblot analysis with anti-GST and anti-MBP antibodies. **g** Overlapping of ECT2- and PAB2-binding targets in *Arabidopsis*. **h** The spatial distance distribution between the PAB2- and ECT2-binding sites.* P*-values were calculated using two-sided Mann–Whitney *U* test. **i** Cumulative distribution of relative mRNA half-life between *ect2/3/4* and WT for Non-targets (black), ECT2 targets (blue), and ECT2 & PAB2 common targets (red). *P*-values were calculated using two-sided Mann–Whitney *U* test

The mechanism by which human m^6^A reader proteins guide and decide RNA fate is determined by their interacting proteins [33]. Thus, to investigate the molecular mechanism of ECT2/ECT3/ECT4-mediated mRNA stabilization, the priority task is to identify their binding proteins. Since ECT2 is the dominant functional protein among ECT2, ECT3, and ECT4, we perform formaldehyde cross-linking and ECT2 immunoprecipitation combined with mass spectroscopy (FA-IP/MS) analysis with and without RNase T1 treatment in *ECT2:ECT2/ect2-1* plants. Note that RNase T1 was used to avoid the RNA-induced protein–protein interaction due to the RNA binding ability of ECT2 (Fig. 4a; Additional file 1: Fig. S12a; Additional file 4: Table S3). Our analysis revealed that poly(A) binding protein 2 (PAB2) and PAB4 were potential interacting proteins of ECT2, regardless of RNase T1 treatment (Fig. 4a; Additional file 1: Fig. S11a;). Additionally, ECT3 was also co-immunoprecipitated with ECT2, providing further evidence for their interaction (Fig. 4a; Additional file 1: Fig. S12a). As PAB family proteins have been shown to promote mRNA stabilization through binding to poly(A) tails in mammals [34–36], we hypothesized that PAB2 and PAB4 could be ECT2’s interacting proteins to facilitate the function of ECT2/ECT3/ECT4-mediated mRNA stabilization. To confirm this, we conducted BiFC and in vitro Y2H assays (Fig. 4b, c; Additional file 1: Fig. S12b, c) which demonstrated direct interactions between PAB proteins and ECT2. Moreover, a correlation analysis of mRNA expression levels revealed that ECT2 had strong co-expression with PAB2 and PAB4 in *Arabidopsis* (Spearman’s *ρ* values between 0.67 and 0.83; Fig. 4d). The plant.MAP database (http://plants.proteincomplexes.org) [37] also supports the stable interaction between ECT2 and PAB family proteins.

To investigate the interaction domain of ECT2 with PAB2 and PAB4, we purified full-length ECT2, as well as four fragments (F1 to F4) containing one or two PrLD domains or YTH domain alone (Fig. 4e), each tagged with GST, and MBP-tagged PAB2 and PAB4 proteins, and conducted in vitro GST pull-down assays to assess the binding of each fragment to PAB2 or PAB4. We found that both PAB2 and PAB4 interacted with full-length ECT2 and each PrLD of ECT2, but not with YTH domain of ECT2 and GST alone (Fig. 4f; Additional file 1: Fig. S12d), suggesting that the PrLDs mediate the physical interaction of ECT2 with PAB proteins.

### PAB2 and PAB4 promote mRNA stabilization in Arabidopsis

Although PAB family proteins have been demonstrated to facilitate mRNA stabilization and translational efficiency through binding to poly(A) tails in mammalian [34, 36, 37], whether PAB2 and PAB4 stabilize mRNA has not been validated in plants [38]. Therefore, we analyzed published CLIP-seq data for PAB2 and PAB4 [38] and our mRNA stability profiling data in WT. We revealed that both PAB2 targets and PAB4 targets (thresholds: IP/Control ≥ 1, *P*-value < 0.05, FPKM value > 1) tended to have longer mRNA half-lives than their Non-targets, confirming the role of PAB2 and PAB4 in mRNA stabilization (Additional file 1: Fig. S13).

### ECT2/ECT3/ECT4 coordinately enhance mRNA stability through recruitment of PAB2 and PAB4

We identified overlapping binding targets of ECT2 and PAB proteins and have found that 61.6% (2268) of ECT2 targets are bound by PAB2 (Fig. 4g) and 50% of ECT2 targets are bound by PAB4 (Additional file 1: Fig. S14a). Further analysis of the spatial distance between their binding regions revealed that the binding sites of PAB2 and PAB4 were in the same region as the ECT2 binding positions (Fig. 4h; Additional file 1: Fig. S14b). By analyzing the mRNA lifetime accumulation between WT and *ect2/3/4* plants, we found that in *ect2/3/4* mutants, the mRNA half-lives of ECT2 & PAB2 common targets were significantly decreased compared to Non-targets (genes not targeted by either ECT2 or PAB2) (Fig. 4i), revealing a co-regulatory function of ECT2 and PAB2 in mRNA stabilization. A similar trend was also observed for ECT2 & PAB4 common targets (Additional file 1: Fig. S14c). Taken together, these results demonstrate the molecular mechanism by which ECT2 binds to m^6^A-modified mRNAs and promotes their stability by directly interacting with PAB2 and PAB4 proteins.

### ECT2/ECT3/ECT4 function in multiple important biological pathways

To further investigate the functions of ECT2/ECT3/ECT4, we analyzed differentially expressed genes in the *ect2/3/4* triple mutant compared with WT using our mRNA-seq data. There were 278 down-regulated and 186 up-regulated genes identified in *ect2/3/4* (FPKM fold change ≥ 2 and* P*-value < 0.05; Additional file 5: Table S4). Gene Ontology (GO) analysis of the 464 differentially expressed genes revealed enrichment in biological processes including response to chitin, cold, wounding, bacterium and fungus, salt and oxidative stresses, abscisic acid, salicylic acid, auxin, and water deprivation (Additional file 1: Fig. S15), suggesting that ECT2/ECT3/ECT4 play regulatory roles in abiotic and biotic stress responses.

### ECT2/ECT3/ECT4 stabilize ABA response-related genes

**Fig. 5:**
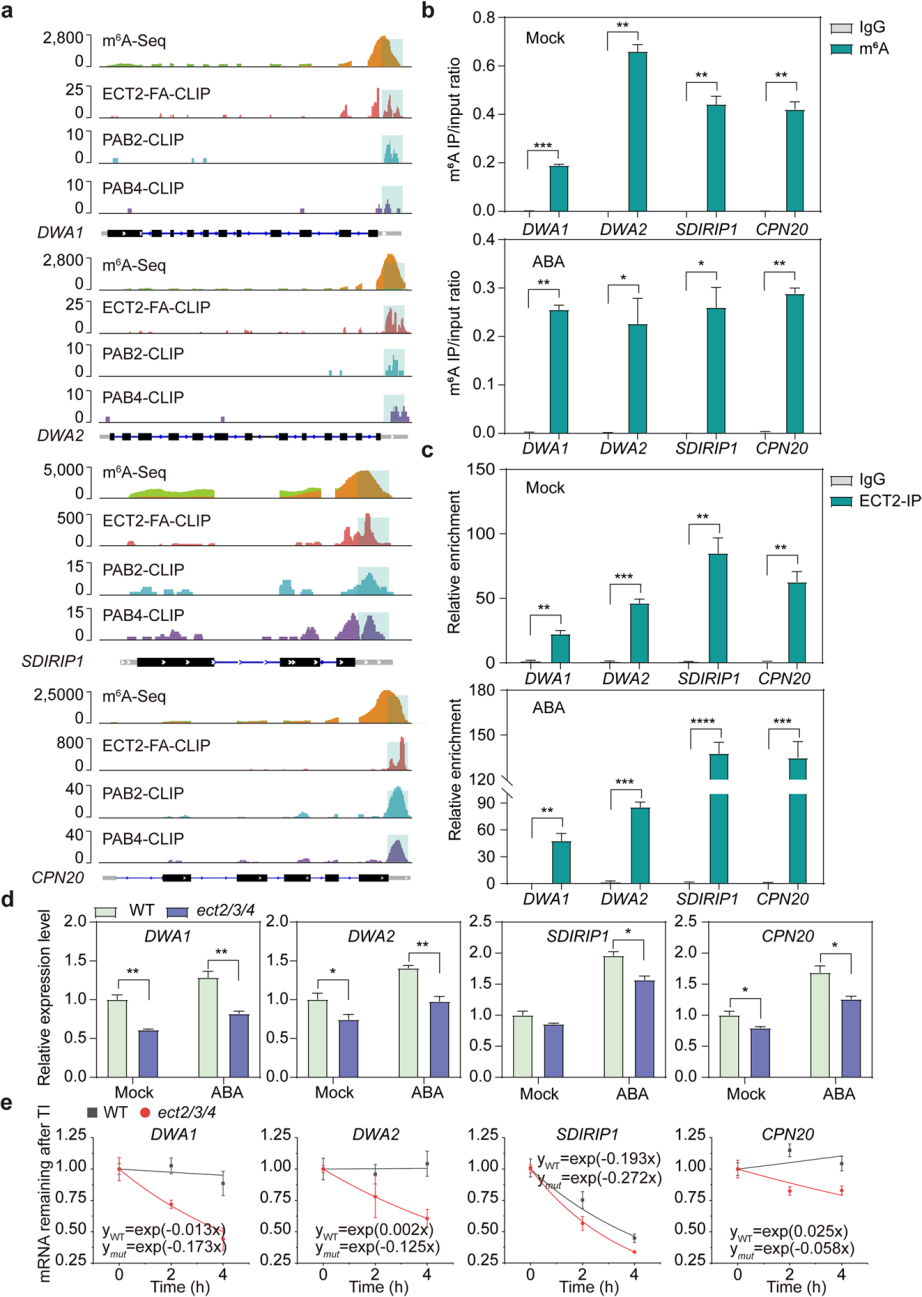
ECT2/ECT3/ECT4 stabilize the ABA response-related genes. **a** Integrative genomics viewer showing the indicated sequencing results on *DWA1*, *DWA2*, *SDIRIP1*, and *CPN20* transcripts. The light blue box labeled in each sequencing result indicated the position of the m^6^A site, the binding sites of ECT2, PAB2, and PAB4. **b** m^6^A-IP-qPCR validation of the m^6^A peaks in *DWA1*, *DWA2*, *SDIRIP1*, and *CPN20* under Mock and ABA treatment. Data are presented as means ± SE, *n* = 2 biological replicates × 2 technical replicates. **c** FA-RIP-qPCR validation of the binding ability of ECT2 towards *DWA1*, *DWA2*, *SDIRIP1*, and *CPN20* in 12-day-old *ECT2:ECT2*/*ect2-1* seedlings under Mock and ABA treatment. Data are presented as means ± SE, *n* = 3 biological replicates × 2 technical replicates. **P < 0.01, ***P < 0.001, ****P < 0.0001 (two-sided *t-*test). **d** Relative mRNA levels of *DWA1*, *DWA2*, *SDIRIP1*, and *CPN20* in 7-day-old WT and *ect2/3/4* seedlings under Mock and ABA treatment. *TUB8* was used as the internal control gene. Data are presented as means ± SE, n = 3 biological replicates × 2 technical replicates. *P < 0.05, **P < 0.01 (two-sided *t-*test). **e** The mRNA lifetime of *DWA1*, *DWA2*, *SDIRIP1*, and *CPN20* in 7-day-old WT and *ect2/3/4* seedlings. TI, transcription inhibition. *18S* was used as the internal control gene. Data are presented as means ± SE, *n* = 3 biological replicates × 2 technical replicates

We then investigated the molecular mechanism underlying ABA hypersensitivity in the *ect2/3/4* mutant. ECT2 can aggregate in cytoplasmic foci in response to heat and drought stresses [15, 16], which may influence its function. Therefore, we first characterized the subcellular localization of ECT2 under 50 μM ABA treatment using *ECT2:ECT2-eGFP/ect2-1* transgenic plants. Confocal images of ECT2-eGFP in 7-day-old *ECT2:ECT2-eGFP/ect2-1* root tips showed that ECT2 was still localized in the cytoplasm and did not aggregate in response to 50 μM ABA treatment (Additional file 1: Fig. S16). This indicated that the ECT2-mediated m^6^A-modified mRNA stabilization regulatory mechanism would not be altered upon ABA stimulation in this experiment. We speculated that ABA signaling-related genes could be modified with m^6^A modification and regulated by the ECT2/ECT3/ECT4-PAB2/PAB4-mediated mRNA stabilization pathway. To test this hypothesis, we selected four ABA signaling-related genes, namely *DWD HYPERSENSITIVE TO ABA* (*DWA*) *1*, *DWA2*, *SDIR1-INTERACTING PROTEIN1* (*SDIRIP1*), and *CHAPERONIN 20* (*CPN20*), from the m^6^A-seq, ECT2-CLIP, PAB2-CLIP, and PAB4-CLIP sequencing results for subsequent mechanistic study. All of these genes are known negative regulators of ABA signaling, and mutants for the genes exhibit enhanced ABA responses such as delayed germination and post-germination development [39–41]. The ECT2-targeted sites at the 3′ UTR of these four genes were highly overlapping with m^6^A sites, PAB2 binding sites, and PAB4 binding sites (Fig. 5a).

We further performed m^6^A-IP-qPCR and FA-RIP-qPCR assays to examine whether *DWA1*, *DWA2*, *SDIRIP1*, or *CPN20* transcripts were modified with m^6^A and whether they were bound by ECT2 under ABA treatment. The m^6^A-IP-qPCR results showed that compared with negative control, *DWA1*, *DWA2*, *SDIRIP1*, and *CPN20* transcripts were consistently modified with m^6^A in 12-day-old control (Mock treatment) or ABA-treated WT seedlings (Fig. 5b; Additional file 1: Fig. S17a), consistent with previously published m^6^A sequencing results from ABA-treated plants [19] (Additional file 1: Fig. S17b). The FA-RIP-qPCR analysis in 12-day-old *ECT2:ECT2/ect2-1* seedlings revealed that endogenous ECT2 bound to *DWA1*, *DWA2*, *SDIRIP1*, and *CPN20* transcripts in both the Mock and ABA treatment conditions; however, the binding ability of ECT2 towards these transcripts was markedly enhanced in ABA stimulation (Fig. 5c). These results confirmed that ECT2 bound to m^6^A-modified *DWA1*, *DWA2*, *SDIRIP1*, and *CPN20* transcripts under both Mock and ABA conditions. We asked whether *DWA1*, *DWA2*, *SDIRIP1*, and *CPN20* transcripts were also bound by PAB proteins. To test this hypothesis, we performed FA-RIP-qPCR assay using the *PAB2:PAB2-Flag* transgenic plants. As expected, *DWA1*, *DWA2*, *SDIRIP1*, and *CPN20* transcripts were also bound by PAB2 in both the Mock and ABA treatment (Additional file 1: Fig. S18), consistent with the reported CLIP-seq results that these four genes are PAB2 and PAB4 targets (Fig. 5a).

We then measured the expression levels of these genes under Mock and ABA treatment using RT-qPCR. *DWA1*, *DWA2*, *SDIRIP1*, and *CPN20* were significantly down-regulated in both Mock and ABA-treated *ect2/3/4* plants (Fig. 5d), consistent with the previously observed enhanced ABA sensitivity phenotype. To investigate the role of mRNA stabilization in regulating these genes, we used actinomycin D to block transcription and measured the mRNA lifetimes of these four genes. The results showed that *DWA1*, *DWA2*, *SDIRIP1*, and *CPN20* transcripts were degraded more rapidly in the *ect2/3/4* mutant than in WT after transcriptional inhibition, whereas the degradation rate of the negative control gene *AT2G07689* was comparable between WT and the *ect2/3/4* mutant (Fig. 5e; Additional file 1: Fig. S19). These results confirmed that m^6^A-modified *DWA1*, *DWA2*, *SDIRIP1*, and *CPN20* transcripts were regulated by an ECT2/ECT3/ECT4-mediated mRNA stabilization pathway.

### ABI5 functions genetically downstream of ECT2/ECT3/ECT4

**Fig. 6:**
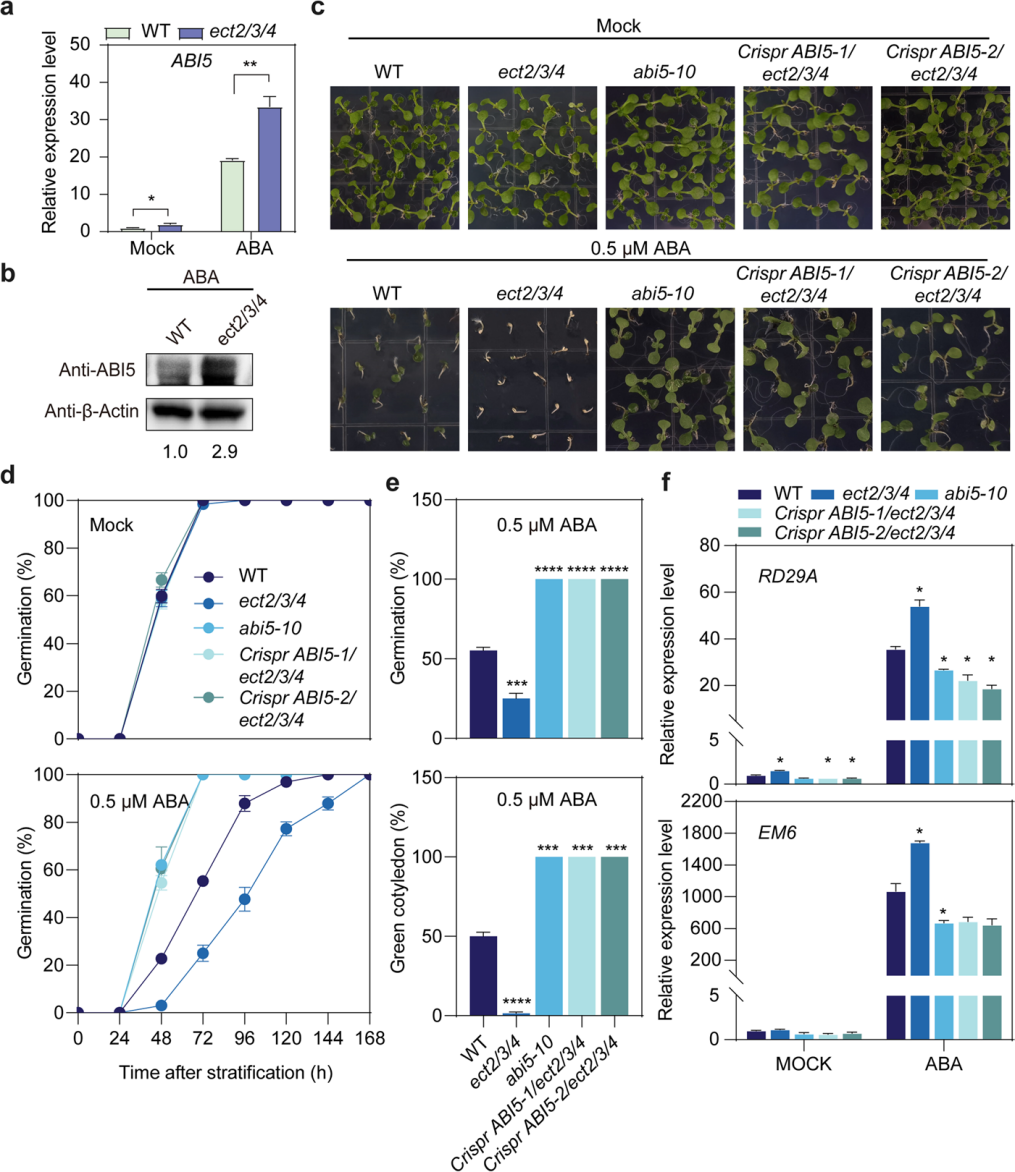
ABI5 functions genetically downstream of ECT2/ECT3/ECT4. **a** Relative mRNA levels of *ABI5* in 7-day-old WT and *ect2/3/4* seedlings under Mock and ABA treatment. *TUB8* was used as the internal control gene. Data are presented as means ± SE, *n* = 3 biological replicates × 2 technical replicates. *P < 0.05, **P < 0.01 (two-sided *t-*test). **b** Western blotting showing the ABI5 protein level in 7-day-old WT and *ect2/3/4* seedlings under ABA treatment. The relative abundance of ABI5 in WT was set to 1 by normalized to the loading control (β-Actin2). **c** Phenotypic analysis of the ABA response in WT, *ect2/3/4*, *abi5-10*, and *Crispr ABI5/ect2/3/4* seeds grown on 1/2 MS-medium supplemented with 0 (Mock) and 0.5 μM ABA under long-day conditions. Representative photographs were taken 8 days after cold stratification. **d** Statistical analysis of germination rates in WT, *ect2/3/4*, *abi5-10*, and *Crispr ABI5/ect2/3/4* plants under ABA treatment. At least 30 seeds per genotype were measured in each replicate. Biological triplicates were averaged. Data are presented as the mean ± SE. **e** Statistical analysis of germination rates 4 days after imbibition and of cotyledon greening rates 8 days after imbibition in WT, *ect2/3/4*, *abi5-10*, and *Crispr ABI5/ect2/3/4* plants under ABA treatment. Data are presented as the mean ± SE; *n* = 3 biological replicates. ***P < 0.001, ****P < 0.0001 compared with WT (two-sided *t-*test). **f** Relative mRNA expression levels of *RD29A* and *EM6* in 7-day-old WT, *ect2/3/4*, *abi5-10*, and *Crispr ABI5/ect2/3/4* seedlings under Mock and ABA treatment. *TUB8* was used as the internal control gene. Data are presented as means ± SE, *n* = 2 biological replicates × 2 technical replicates. *P < 0.05 compare with WT (two-sided *t-*test)

*ABA INSENSITIVE5* (*ABI5*) is a downstream gene that is negatively regulated by DWA1, DWA2, SDIRIP1, and CPN20 [39–41]. Elevated levels of ABI5 are associated with germination repression and post-germination developmental arrest. Consequently, it is plausible that the ABA hypersensitivity of *ect2/3/4* was mediated entirely by up-regulation of ABI5. To test this possibility, we measured ABI5 mRNA and protein levels in 7-day-old WT and *ect2/3/4* seedlings under ABA treatment. Both mRNA and protein levels of ABI5 were significantly increased in the *ect2/3/4* mutant after 50 μM ABA treatment (Fig. 6a, b). Next, we examined the genetic role between ABI5 and ECT2/ECT3/ECT4 by generating the two ABI5 CRISPR knockout lines in *ect2/3/4*, namely *Crispr ABI5-1/ect2/3/4* and *Crispr ABI5-2/ect2/3/4* quadruple mutants*.* Two mutant lines were confirmed as homozygous mutants by Sanger sequencing (Additional file 1: Fig. S20). Germination assays showed that all mutant seeds were indistinguishable from WT seeds under normal condition. However, in the presence of ABA, *Crispr ABI5-1/ect2/3/4* and *Crispr ABI5-2/ect2/3/4* mutants were both insensitive to ABA for seed germination and post-germination growth, similar to *abi5-10* but different from *ect2/3/4* (Fig. 6c–e; Additional file 1: Fig. S21). These results indicate that ABI5 functions genetically downstream of ECT2/ECT3/ECT4.

ABI5 is known to transactivate *RESPONSIVE TO DESICCATION 29A* (*RD29A*) and *EARLY METHIONINE-LABELED 6* (*EM6*) [42, 43]; we therefore examined *RD29A* and *EM6* expression among WT, *ect2/3/4*, *abi5-10*, and *Crispr ABI5/ect2/3/4* mutant plants treated with or without ABA. RT-qPCR results showed that compared to WT, *RD29A* and *EM6* were significantly upregulated in the *ect2/3/4* mutant and downregulated in both *abi5-10* and *Crispr ABI5/ect2/3/4* mutants under 50 μM ABA treatment (Fig. 6f). Thus, the results indicate that ABI5 up-regulation in the *ect2/3/4* mutant contributed to increased ABA sensitivity via up-regulation of downstream ABA-responsive genes.

### CPSF30-L and ECT2/ECT3/ECT4 bind to some of the same m^6^A sites and execute distinct RNA fate regulation

**Fig. 7:**
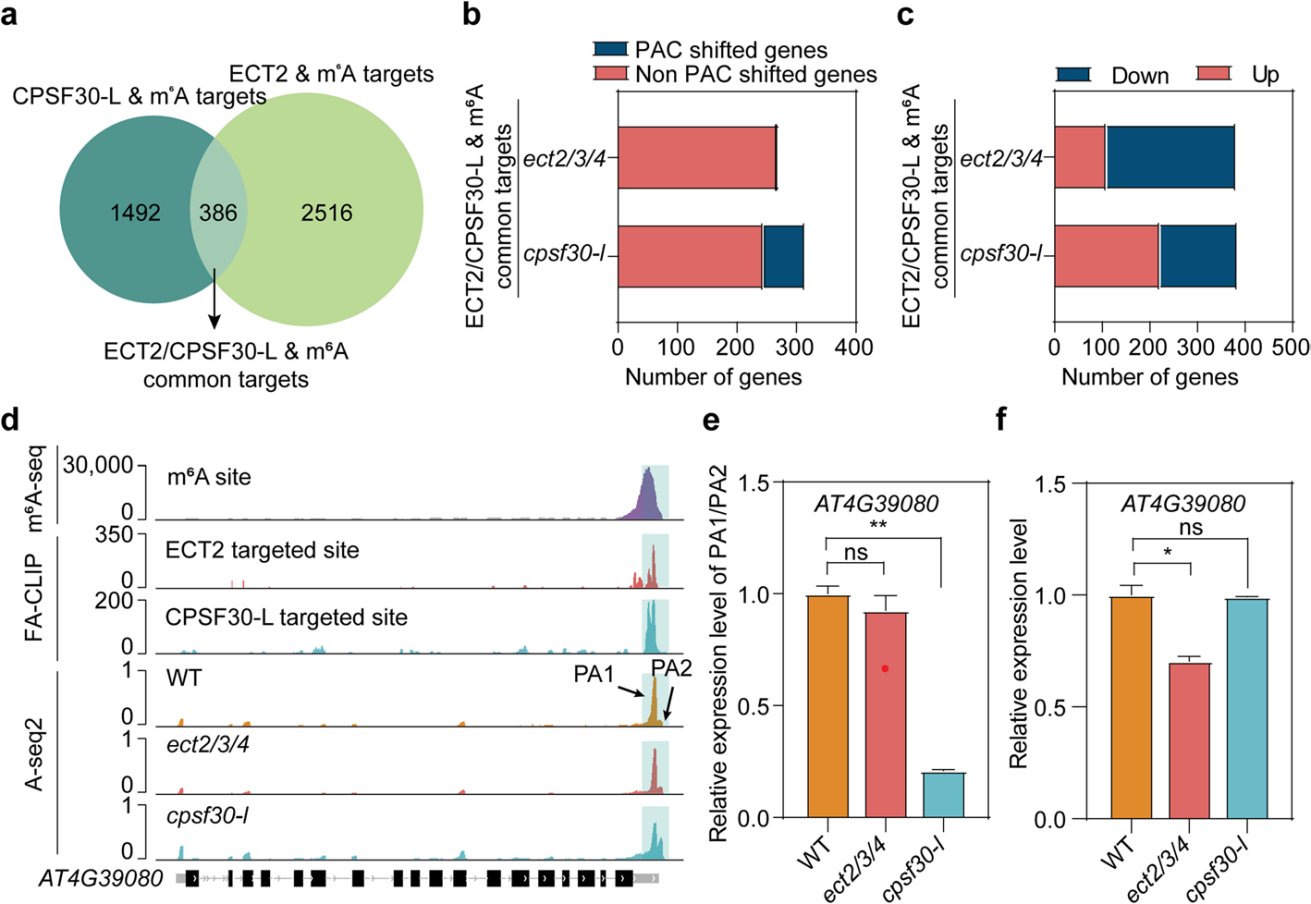
CPSF30-L and ECT2/ECT3/ECT4 bound some of the same m^6^A sites and execute distinct RNA fate regulation. **a** Overlap of the identified CPSF30-L & m^6^A targets and ECT2 & m^6^A targets corresponding to 386 unique transcripts with the same m^6^A site (termed ECT2/CPSF30-L & m^6^A common targets). **b** Bar plots showing the amount of PAC shifted and Non-PAC shifted genes in ECT2/CPSF30-L & m^6^A common targets in *ect2/3/4* and *cpsf30-l* mutants, respectively. **c** Bar plots showing the amount of upregulated and downregulated genes in ECT2/CPSF30-L & m^6^A common targets in *ect2/3/4* and *cpsf30-l* mutants, respectively. **d** Integrative genomics viewer showing sequencing results for *AT4G39080* transcripts. The light blue box at the far right of each line indicates the position of the m.^6^A site, ECT2 and CPSF30-L binding sites, and the position of the shifted poly(A) site. PA1 and PA2 are indicated as proximal and distal poly(A) sites. The *y*-axis scales were normalized by the mapped sequencing reads. **e** Relative expression of proximal and distal transcripts produced by PA1 and PA2 in WT, *ect2/3/4*, and *cpsf30-l* plants. **f** Relative mRNA level of *AT4G39080* in WT, *ect2/3/4*, and *cpsf30-l* plants. *TUB8* was used as the internal control gene. Data are presented as the mean ± SE; *n* = 2 biological replicates × 2 technical replicates. *P < 0.05, **P < 0.01 (two-sided *t-*test)

CPSF30-L is another m^6^A reader that regulates poly(A) site choice in nuclear bodies [18]. To further investigate the distinctive regulatory mechanism of two different types of m^6^A readers that recognize the same m^6^A site, we compared CPSF30-L & m^6^A targets with ECT2 & m^6^A targets and identified 386 ECT2/CPSF30-L & m^6^A common targets (i.e., mRNAs that contain the same m^6^A peak bound by both ECT2 and CPSF30-L; Fig. 7a). Of the PAC detected ECT2/CPSF30-L & m^6^A common targets, 22.15% of the genes (70 out of 316) had a significant PAC shift in *cpsf30-l* mutant, but fewer genes (2 out of 271) had altered poly(A) sites in *ect2/3/4* compared with WT (Fig. 7b). In addition, nearly 70% of the ECT2/CPSF30-L & m^6^A common targets were down-regulated in the *ect2/3/4* mutant but not in the *cpsf30-l* mutant compared to WT (Fig. 7c). To better distinguish the specialized effects of CPSF30-L and ECT2/ECT3/ECT4 on m^6^A-modified mRNAs, we selected one gene from ECT2/CPSF30-L & m^6^A common targets, *AT4G39080*, as a representative case. A-seq2 sequencing results showed that the proximal poly(A) site (PA1) of the *AT4G39080* transcript in WT was shifted to the distal poly(A) site (PA2) in *cpsf30-l* but not *ect2/3/4* mutants (Fig. 7d). RT-qPCR was then used to measure the relative expression levels of PA1 and PA2 in WT, *ect2/3/4*, and *cpsf30-l* plants. Compared with WT, the relative expression ratio of PA1/PA2 was dramatically decreased in *cpsf30-l*, but there was no difference in the *ect2/3/4* mutant (Fig. 7e). Additionally, we also found that *AT4G39080* transcript levels were significantly decreased in *ect2/3/4* compared to WT (Fig. 7f).

**Fig. 8:**
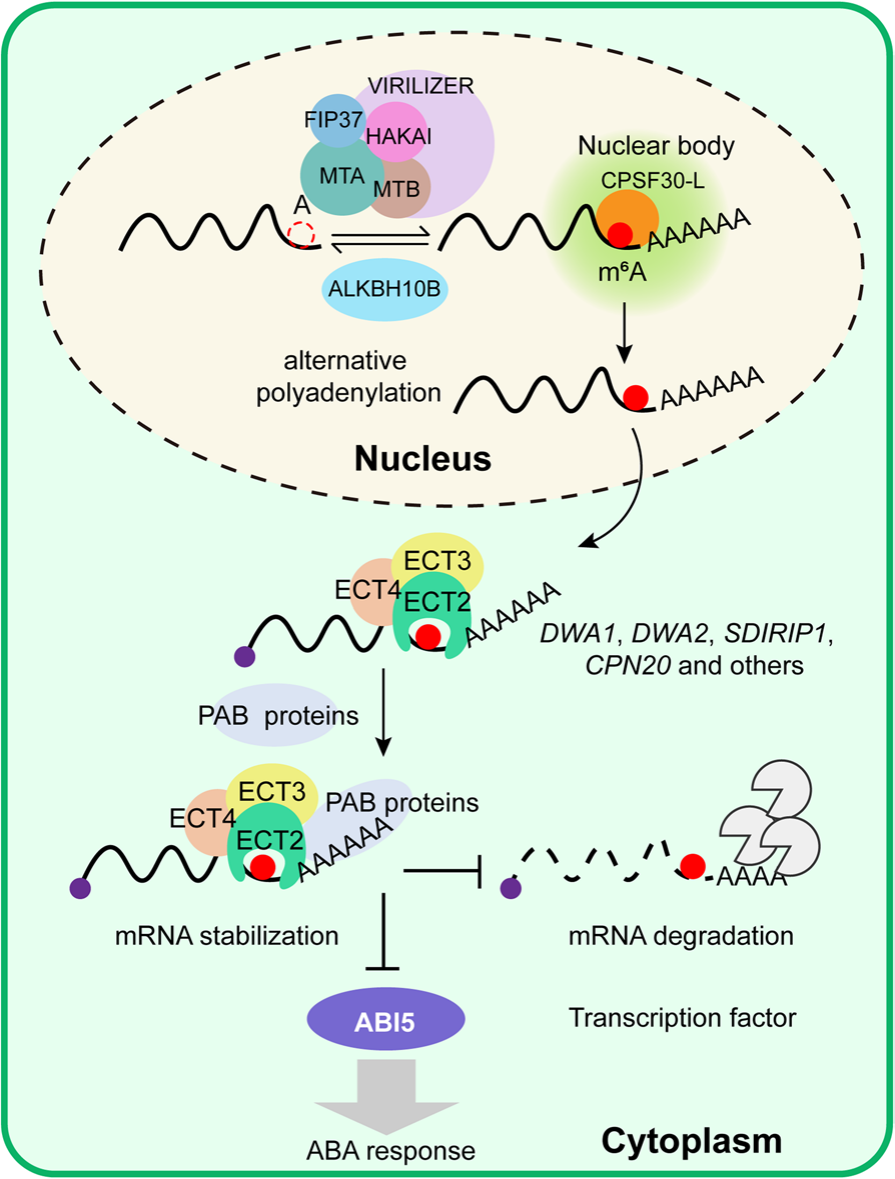
A working model for the regulatory roles of m^6^A readers CPSF30-L and ECT2/ECT3/ECT4. In the nucleus, CPSF30-L binds m^6^A-modified transcripts and regulates its poly(A) site choice; in the cytoplasm, m^6^A readers ECT2, ECT3, and ECT4 interact each other, where ECT2/ECT3/ECT4 bind m^6^A-modified transcripts tightly and recruit the poly(A) binding proteins through the direct interaction between ECT2 and PAB2/PAB4, thereby preventing deadenylation and stabilizing their targeted mRNA. In ABA response, ECT2/ECT3/ECT4 redundantly maintain mRNA stability of ABA response-related genes (e.g., *DWA1*, *DWA2*, *SDIRIP1*, and *CPN20*), thereby repressing the accumulation of ABI5 and regulating ABA-mediated seed germination and post-germination growth

Collectively, our findings demonstrate the distinct regulation of gene expression by CPSF30-L and ECT2/ECT3/ECT4. The m^6^A-modified RNAs was bound by CPSF30-L for APA regulation in the nucleus, but in the cytoplasm, ECT2, ECT3, and ECT4 form a complex through direct protein–protein interactions, and enhance the stability of its targets in an m^6^A-dependent manner. Mechanistically, ECT2 directly recruits PAB2 and PAB4 proteins and coordinately maintains their cognate mRNA stabilization. Upon ABA stimulation, deficiency of *ECT2/ECT3/ECT4* destabilizes *DWA1*, *DWA2*, *SDIRIP1*, and *CPN20* transcripts, promoting the accumulation of ABI5 and regulating ABA-mediated seed germination and post-germination growth (Fig. 8).

## Discussion

m^6^A RNA modification is an essential epitranscriptomic mark that regulates transcriptional and post-transcriptional gene regulation. Recently, engineering m^6^A marks by overexpression of human m^6^A demethylase fat mass and obesity-associated protein (FTO) in rice and potato was found to dramatically boost field yield and biomass, highlighting the modulation of plant m^6^A as a promising direction for crop breeding [25]. m^6^A reader proteins have the capacity to recognize m^6^A modifications, allowing them to facilitate the gene regulatory functions of m^6^A. Thus, understanding the molecular mechanisms of m^6^A reader-mediated m^6^A function in gene regulation could provide opportunities for breeding crops with better agronomic traits. To date, only four *Arabidopsis* m^6^A readers, ECT2, ECT3, ECT4, and CPSF30-L, have been identified [14–18]. Genetic experiments have demonstrated functional redundancy of ECT2, ECT3, and ECT4, but the specific biological role of ECT2/ECT3/ECT4 in RNA processing has remained unknown. Furthermore, while ECT2 was found to promote m^6^A-modified mRNA stabilization [14], it remained unclear whether and how ECT2/ECT3/ECT4 stabilizes m^6^A-modified mRNA at the molecular level. Here, we discovered that ECT2, ECT3, and ECT4 interact with each other in the cytoplasm, which leads to their functional redundancy. ECT2, ECT3, and ECT4 cooperatively bind target transcripts and promote m^6^A-modified mRNA stabilization through interactions with the poly(A)-binding proteins PAB2 and PAB4. Disruption of *ECT2/ECT3/ECT4* leads to ABA hypersensitivity through destabilization of mRNAs for ABA signaling-related genes.

Although ECT2, ECT3, and ECT4 are homologs of human YTHDF family proteins, their functions entirely differ from the human homologs YTHDF2 or YTHDF1. Human YTHDF2 promotes m^6^A-modified mRNA degradation through interactions with the CCR4-NOT deadenylase complex [5, 33], and human YTHDF1 facilitates mRNA translation efficiency by interacting with eukaryotic initiation factor complex 3 (EIF3) [6]. In contrast, we found that ECT2, ECT3, and ECT4 promote m^6^A-modified mRNA stabilization through binding with PAB family proteins (Fig. 4), thereby protecting the poly(A) tail from deadenylation. ECT2, ECT3, and ECT4 have no function in translation (Additional file 1: Fig. S10e, f). The differing roles of *Arabidopsis* ECT2/ECT3/ECT4 and mammalian YTHDF2 highlight an additional layer of diversity and complexity in m^6^A functions between different species. The distinct regulatory effect is largely modulated by the interacting protein partners of m^6^A readers between plants and mammals. Thus, predicting the biological function of plant m^6^A readers based on the roles of mammalian m^6^A readers would be challenging. It is necessary to identify and decipher the molecular functions of plant m^6^A readers.

PAB2 and PAB4 were reported to enhance translation efficiency rather than mRNA stability [38]. Here we analyzed the mRNA half-life of the identified PAB2- and PAB4-targeted RNA from the reported CLIP-seq using our mRNA stability sequencing results showed that PAB2 and PAB4 promote mRNA stabilization (Additional file 1: Fig. S12). To seeking for the reason, we found the difference is that we added ERCC RNA spike-in control in total RNA samples, which will give better RNA quantification and reduce the sample variation. Although PAB2 and PAB4 have dual functions in promoting translation efficiency and mRNA stability, we found that the number of overlapping targets between ECT2 and PAB2 or PAB4 were around 27% of total PAB2 or PAB4 targets (Fig. 5g; Additional file 1: Fig. S13). This might explain why ECT2/ECT3/ECT4 recruits PAB2 and PAB4 to promote mRNA stabilization, not translation efficiency.

Our previous ECT2 FA-CLIP-seq results showed that ECT2-binding RNA sites are enriched around the FUE region of the polyadenylation signal, suggesting that ECT2 may play a role in APA regulation. Through transcriptome-wide poly(A) site sequencing and single gene validation assays in WT and *ect2/3/4* mutant plants, we demonstrated that ECT2/ECT3/ECT4 have no function in alternative polyadenylation (Additional file 1: Fig. S9a-d), consistent with their localization to the cytoplasm [16] (Additional file 1: Fig. S7). CPSF30-L is the only established nuclear m^6^A reader that regulates alternative polyadenylation [18]. Although *ect2/3/4* and *cpsf30-l* mutants showed similar ABA hypersensitivity phenotypes, they are two distinct types of m^6^A readers that regulate ABA signaling-related genes through different m^6^A-mediated pathways.

A central question for understanding m^6^A function is whether two different m^6^A readers can bind the same m^6^A modification position on the same mRNA and perform different regulatory functions in RNA processing. Intriguingly, the methylated *AT4G39080* transcript bound by both CPSF30-L and ECT2 demonstrated distinctive regulatory functions by the different m^6^A reader proteins. *AT4G39080* encodes vacuolar proton ATPase A3 (VHA-A3), a crucial component of the tonoplast V-ATPases that regulates nutrient storage [44]. Disruption of *CPSF30-L* affected alternative polyadenylation of *VHA-A3* pre-mRNA, leading to a higher proportion of transcripts with distal poly(A) sites (Fig. 7e), whereas disruption of *ECT2/ECT3/ECT4* destabilized *VHA-A3* mRNA (Fig. 7f). We found that the m^6^A reader CPSF30L bound *VHA-A3* pre-mRNA to regulate APA in the nucleus and that m^6^A readers ECT2/ECT3/ECT4 bound *VHA-A3* mRNA for stabilization. Thus, the same m^6^A sites can be recognized and modulated by two different types of m^6^A reader proteins.

We further demonstrated that ECT2/ECT3/ECT4 redundantly and negatively regulate ABA signaling during seed germination and post-germination growth, with ECT2 playing a core regulatory role, based on the finding that ABA hypersensitivity is less severe in *ect3/4* than in *ect2/4* mutants (Fig. 2). Although ECT2 is the most abundant m^6^A reader in *Arabidopsis*, it requires ECT3 and ECT4 to perform the stabilization function for m^6^A-modified mRNA. Our comprehensive mechanistic study can explain previous findings, e.g., that ECT2 and ECT3 are required for normal trichome branching [16]; ECT2 interacts with ECT3 and cooperatively bind m^6^A-modified mRNAs related to trichome morphogenesis, such as *TTG1*, *ITB1*, and *DIS2*, which have been reported as ECT2 targets [14] for mRNA stabilization. In most cases, ECT2, ECT3, and ECT4 redundantly regulate biological processes, such as ABA signaling and leaf development [16]. In these cases, ECT2, ECT3, and ECT4 bind the same m^6^A-modified mRNA tightly and promotes mRNA stabilization by recruiting PAB proteins (PAB2 and PAB4). The PAB proteins protect the poly(A) tail of ECT2/ECT3/ECT4 targets from deadenylation. GO functional analysis revealed that ECT2, ECT3, and ECT4 may play redundant regulatory roles in responses to biotic stresses (fungus and bacterium) and abiotic stresses (such as cold, salt, and salicylic acid).

## Conclusions

In summary, our study demonstrated that the m^6^A readers ECT2, ECT3, and ECT4 tightly interact with each other and bind to m^6^A-modified mRNA, promoting stabilization of target mRNAs through recruitment of PAB proteins. The spatial coordination of ECT2, ECT3, and ECT4 regulates the m^6^A-mediated stabilization pathway, leading to genetic redundancy, as evidenced by the observed phenotypes. This novel model sheds light on the complex role of multiple m^6^A readers in mediating m^6^A function in plants.

## Methods

### Plant material and growth conditions

*ect2-1* (SALK_002225), *ect3-2* (GABIseq487H12.1), *ect4-1* (SALK_151516), and *abi5-10* (SALK_200891) mutant lines were in the *Arabidopsis thaliana* Col-0 ecotype background and obtained from the Arabidopsis Biological Resource Center (ABRC). All seeds of WT and mutants were sterilized in 75% ethanol for 10 min followed by immersion in 20% bleaching solution for additional 10 min, and immediately rinsed at least four times with sterile water. The sterilized seeds were stratified at 4 °C in darkness for 3 days and grown on 0.5 × Murashige and Skoog (1/2 MS) nutrient agar plates for 12 days and then transferred to soil. All plant germination and growth were under long-day conditions (16 h light/8 h dark at 22 °C with a light intensity of 90 to 120 μmol m^−2^ s^−1^).

### Generation of Crispr *ABI5/ect2/3/4* mutant by the CRISPR/Cas9 system

The *Crispr ABI5/ect2/3/4* mutants were obtained following the published protocol [45]. In brief, single guide RNA (sgRNA) sequences of *ABI5* were amplified by PCR with pDT1T2 vector as template. The purified product was ligated into a binary vector pHEE401E. The constructed plasmid was transformed into *ect2/3/4* mutant via floral dipping method. The positive seedlings were screened from 1/2 MS plates with hygromycin B and identified with Sanger sequencing.

### ABA phenotypic analysis and ABA treatment

All different genotypic plants were grown in the same conditions, and their seeds were collected at the same time. The mature seeds were dried and stored at room temperature. ABA phenotypic experiments were repeated at least three times. Three replicates (> 40 seeds per genotype) were conducted on 1/2 MS medium supplemented with various concentrations of ABA (Sigma-Aldrich). Germination (emergence of radicles) and post-germination growth (green cotyledon appearance) were scored at regular intervals, respectively. For ABA treatment assays, 7-day-old seedlings grown 1/2 MS agar plates were transferred to 1/2 MS-liquid medium supplemented with 50 μM ABA or not for 3 h.

### RT-qPCR

Isolated RNAs were reverse transcribed into the first strand cDNA by using SuperScript III (Thermo Fisher Scientific). The transcribed cDNAs were diluted into an appropriate concentration and used as templates to perform PCR reactions with Hieff ® qPCR SYBR Green Master Mix (Low Rox) (Yeasen). These reaction systems were then analyzed on the ViiATM7 instrument (Applied Biosystems) according to the instruction. To ensure the accuracy of results, *TUB 8* acts as an internal control and each independent sample contains at least three biological replicates and two technical replicates. All used primers are listed in Additional file 6: Table S5.

### Subcellular localization

Root tips of 7-day-old *ECT2:ECT2-eGFP* transgenic seedlings were used to examine the subcellular localization of endogenous ECT2 protein under ABA or Mock treatment. Analysis of subcellular localization was performed on LSM700 (Zeiss) confocal laser scanning microscope using a 63 × oil objective. We used 488 nm wavelength laser to excite the eGFP and collected the emission signal from 485 to 530 nm.

### Protein expression and purification

The plasmids containing GST, GST-ECT2 fragments, MBP-PAB2, or MBP-PAB4 were transfected into *Escherichia coli* strain BL-21 Gold competent cells. Protein expression was induced at 18℃ with 500 nM IPTG for 16 h. Cells were collected and resuspended in lysis buffer (10 mM Tris–HCl, pH 8.0, 500 mM NaCl, 1 mM PMSF, 3 mM DTT, and 5% glycerol) and then lysed by sonication and centrifuged. The soluble GST-tagged proteins were purified with GST affinity column (GE Healthcare) and eluted by using elution buffer (10 mM Tris–HCl, pH 8.0, 500 mM NaCl, 10 mM reduced glutathione, and 3 mM DTT). The soluble MBP-tagged proteins were purified with amylose resin (NEB). These purified proteins were stored in storage buffer (10 mM Tris–HCl, pH 8.0, 200 mM NaCl, 1 mM DTT, and 20% glycerol) at − 80℃.

### Yeast two-hybrid (Y2H) assays

For Y2H assays, the full-length ECT2, ECT3, ECT4, PAB2, and PAB4 coding sequence were sub-cloned into the pGBKT7 (for GAL4 BD fusion) and pGADT7 (for GAL4 AD fusion) vectors. The recombinant constructs were co-transformed into AH109 cells. The transformed cells were grown on double dropout medium deficient in -Leu/-Trp, and protein interactions were assessed on triple or quadruple dropout medium deficient in -Leu/-Trp/-Ade, -Leu/-Trp/-His, and -Leu/-Trp/-Ade/-His.

### Bimolecular florescence (BiFC) assays

The full-size ECT2, ECT3, ECT4, PAB2, and PAB4 coding sequence were fused inframe to the 5′ end of a gene sequence encoding the C-terminal half of YFP in the pBI121-cYFP vector or the N-terminal half of YFP in the pBI121-nYFP vector. The recombinant construct was transfected into Agrobacterium GV3101 (ZOMANBIO) by the freeze–heat shock method. Pairwise combinations were co-infiltrated into 4-week-old *Nicotiana benthamiana* leaves. P19 was used to inhibit transgenic silencing. Infiltrated *Nicotiana benthamiana* leaves were first incubated at 23 °C for 24 h in darkness. The YFP signal was observed after 48–60 h of infiltration using a LSM700 (Zeiss) confocal laser scanning microscope with a 20 × objective.

### In vitro pull-down assay

Purified MBP-PAB2 or MBP-PAB4 protein (100 pmol) was incubated with 25 μl of pierce glutathione magnetic agarose beads to be pre-cleared in 200 µL IPP buffer (150 mM NaCl, 0.1% NP-40, 10 mM Tris, pH 7.4, 0.5 mM DTT) for 1 h with gentle rotation at 4 °C. Then, the pre-cleared MBP-PAB2 or MBP-PAB4 protein was mixed with 100 pmol GST or GST-ECT2 fragments for 2 h at 4 °C with gentle rocking and equal amount of pierce glutathione magnetic agarose beads. The beads were then washed five times with 0.5 ml binding buffer and proteins were eluted by boiling the beads with 40 μl SDS loading buffer at 95 °C for 10 min. Proteins were analyzed by 12% SDS–PAGE followed by western blotting analysis with anti-MBP (Mei5bio) and anti-GST (Genscript).

### Formaldehyde cross-linking and immunoprecipitation combined with MS analysis.

FA-IP/MS assay was based on the previously described FA-CLIP method [14]. 12-day-old *ECT2:ECT2/ect2-1* seedlings were harvested and fixed in 80 mL of 1% formaldehyde solution under vacuum for 15 min at room temperature; 5 mL 2 M glycine solution was added to quench the cross-linking reaction for additional 5 min under vacuum. The fixed samples were washed three times with pre-cooled water and immediately frozen in liquid nitrogen; 2 g fixed plant material for each sample was ground into powder and incubated into 2 mL lysis buffer [150 mM KCl, 50 mM HEPES, pH 7.5, 2 mM EDTA, 0.5% NP-40 [*v*/*v*], 1 × cocktail protease inhibitor, and 40 U/mL RNase inhibitor] with rotation at 4 ℃ for 30 min. The lysates were centrifuged at 15,000 rpm for 30 min at 4 °C and filtered through a 0.22-μm membrane syringe. Turbo DNase (2 U/mL; Thermo Fisher Scientific) and RNase T1 (1000 U/mL; Thermo Fisher Scientific) were added into each sample for 15 min at 22 °C. The lysates were subsequent immunoprecipitated with pre-washed Anti-Flag M2 beads (Sigma-Aldrich) or a control IgG conjugated with protein A Dynabeads on a rotating wheel for 4 h at 4 °C. The beads were collected and washed sequentially four times with wash buffer [150 mM KCl, 50 mM HEPES, pH 7.5, 0.05% NP-40 [*v*/*v*], 40 U/mL RNase inhibitor, and 1 × cocktail protease inhibitor], followed with RNase T1 treatment (20 U/μL) for 20 min at 22 °C. The immunoprecipitates were eluted with wash buffer supplemented with 500 ng/μL 3 × Flag peptide overnight. Eluates were gel-purified and subjected to mass spectrometry analysis.

### mRNA-seq

Total RNA of 12-day-old seedlings was extracted using TRIzol reagent (Invitrogen), and RNA integrity was assessed with RNA integrity number (RIN) using Agilent 2100 system following the manufacturer’s instructions; 5 μg intact total RNA was used to isolate the poly(A)^+^ RNA using oligo(dT)_25_ Dynabeads (Thermo Fisher Scientific) for each sample. Library construction was prepared using NEB Next Ultra II RNA Library Prep Kit (NEB), and sequencing was performed on an Illumina HiSeq X Ten machine in pair-end mode with 150 bp per read (Genewiz).

### mRNA lifetime sequencing

7-day-old WT and *ect2/3/4* seedlings grown on 1/2 MS medium were treated with 200 μM actinomycin D (Sigma-Aldrich) and were collected at 0, 4, and 6 h. Ten seedlings were harvested in duplicates and immediately frozen in liquid nitrogen. The total RNA was extracted by Trizol reagent (Invitrogen) and accessed its RNA integrity using Agilent 2100 system for subsequent RNA-seq. For RNA-Seq, an equal amount of external RNA control consortium (ERCC) RNA spike-in control (Thermo Fisher Scientific) was added to the total RNA samples as internal controls. The RNA was subjected to Dynabeads mRNA Purification Kit (Thermo Fisher Scientific) followed by library construction using NEB Next Ultra II RNA Library Prep Kit (NEB). Sequencing was performed on an Illumina HiSeq X Ten machine in pair-end mode with 150 bp per read (Genewiz).

### Ribosome profiling

12-day-old WT, *ect2-1*, and *ect2/3/4* seedlings were harvested and immediately frozen in liquid nitrogen. About 1 g of well-ground seedlings was resuspended in 1 mL of pre-cold polysome extraction buffer [200 mM Tris–HCl pH 8, 50 mM KCl, 25 mM MgCl_2_, 2% (vol/vol) polyoxyethylene (10) tridecyl ether, 1% deoxycholic acid, 2 mM DTT, 100 μg/mL cycloheximide, and 10 U/mL DNase I], and rotated continuously for 30 min at 4 °C. The resuspended extracts were spun at 15,000 rpm at 4 °C for 30 min and filtered through a 0.22-μm membrane syringe; 200 μL of the resulting supernatant were saved as input sample, the other 800 μL solution were treated with MNase digestion for 15 min at 22 °C, and then quenched by adding 20 U of SUPERase-in (Thermo Fisher Scientific). The digested samples were loaded on a pre-cold 10–50% (wt/vol) sucrose gradient [40 mM Tris–HCl pH 8.4, 20 mM KCl, 10 mM MgCl_2_, and 5 μg/mL cycloheximide] were spun in a SW-40Ti rotor (Beckmann) at 27,500 rpm for 4 h at 4 °C and then fractionated using a BioRad EM-1 Econo UV monitor. 80S fraction was collected to extract RNA. The isolated RNA was separated by 15% (wt/vol) TBE-urea PAGE (Thermo Fisher Scientific), and gel slices from 28 to 30 nt were excised. Ribosome footprints were recovered from the excised gel slices, and then was subjected to 3′ dephosphorylation and 5′ phosphorylation followed by library construction using NEBNext Multiplex Small RNA Library Prep Kit for Illumina (NEB). Sequencing was performed on an Illumina HiSeq X Ten machine in pair-end mode with 150 bp per read (Genewiz).

### Polyadenylation site profiling

A-seq2 was performed for two independent biological replicate samples from *ect2/3/4* and WT as described previously [46]. Briefly, 500 ng poly(A)^+^ RNA from 12-day-old seedlings per sample was purified using and fragmented in alkaline fragmentation buffer for 7 min at 95 °C and 650 rpm. After 5′ end phosphorylation and DNase treatment, the 3′ ends fragmented RNA was blocked by incubating with cordycepin triphosphate at 37 °C for 30 min. revRA-3′ adaptor was ligated to 5′ ends RNA at 24 °C for additional 16 h. Reverse transcription was carried out using Biotin-dU-(dT)_25_. The first-strand cDNA was enriched by Streptavidin beads (Invitrogen) and then isolated by USER (NEB) and RNase H (NEB) digestion. After ligation of revDA-5′ to the 5′ ends of cDNA, the generated cDNA was amplified using NEBNext® Multiplex Oligos (NEB) for 12 cycles. PCR products were separated on a 5% TBE gel, and 180- to 300-bp bands were excised and purified. Sequencing was performed on an Illumina HiSeq X Ten machine in pair-end mode with 150 bp per read (Genewiz). All oligos are listed in Additional file 6: Table S5.

### *m*^*6*^*A-IP-qPCR*

m^6^A-IP-qPCR was performed as previously described [18]. As for one sample, 20 ng poly(A)^+^ RNA was saved as input RNA and 400 ng poly(A)^+^ RNA without fragmentation was incubated with 1 μg m^6^A antibody (Synaptic Systems) in a head-over-tail rotation for 2 h at 4 °C. The m^6^A-containing fragments were then immunoprecipitated with 10 μL pre-cleared Protein A Dynabeads (Thermo Fisher Scientific) for 2 h on a rotating wheel at 4 °C and then eluted with 6.7 mM m^6^A-containing buffer twice. After ethanol precipitation, both m^6^A-bound RNA fraction and input RNA were reverse transcribed and calculated the enrichment fold for specific transcripts by qPCR assay. *AT2G07689* was used as the internal control gene.

### In vivo FA-RIP-qPCR

The FA-RIP-qPCR assay was performed following a previously described procedure [14]. Briefly, 7-day-old Mock or ABA treated *ECT2:ECT2/ect2-1* seedlings were separately fixed with 1% formaldehyde solution. The fixed plant materials were ground into powder and incubated with lysis buffer in a head-over-tail rotation for 30 min at 4 °C. After fully lysis and centrifugation, the lysates were collected and then immunoprecipitated with Anti-Flag M2 beads (Sigma-Aldrich; Flag-IP) or a control IgG conjugated with protein A Dynabeads (IgG-IP) on a rotating wheel for 2 h at 4 °C. After washing, proteinase K digestion and ethanol precipitation, the recovered RNA fractions were reverse transcribed into cDNA to calculate the relative enrichment fold via RT-qPCR. *AT2G07689* was used as the internal control.

### mRNA stability assay

7-day-old WT and *ect2/3/4* seedlings were transferred to 10-cm Petri dishes containing 10 mL 1/2 MS medium supplemented with 200 μM actinomycin D. Seedlings were collected and referred as time 0 h control and subsequent samples were harvested at 2 and 4 h, respectively. Three biological replicates were performed at indicated time points with a pooling of ~ 10 plants for each replicate. RT-qPCR assays were conducted to access the degradation rate of targeted transcripts. 18S RNA was used as the reference gene. *AT2G07689* was used as a negative control. *y* = exp (-A × x) equation was used to calculate the decay rate.

### Analysis of mRNA-seq data

Sequencing reads were trimmed using Cutadapt (v1.18) to remove adaptor. Clean reads were next mapped to the reference genome (TAIR10) [47] by HISAT2 (v2.1.0) [48]. FPKM values were calculated with StringTie (v1.3.5) [49]. The gene expression pattern between WT and *ect2-3–4* was analyzed by R package (Ballgown) [50], and genes with FPKM fold change ≥ 2 and *P-*value < 0.05 were regarded as differentially expressed genes.

### Analysis of mRNA lifetime-seq data

Adaptor sequences of raw reads were trimmed by Cutadapt (v2.7), and the remaining reads were mapped to the *Arabidopsis* genome (TAIR10) [47] using HISAT2 (v2.2.1) [48]. After removal of PCR duplications with MarkDuplicates function of Picard software, the remaining reads were normalized to the linear-fitting of RNA spike-in to calculate RPKM values [51]. Genes with normalized RPKM value > 1 were selected for the next calculation of degradation rate and half-life. As actinomycin D treatment results in transcription stalling, the change of mRNA concentration at a given time (dC/dt) is proportional to the constant of mRNA decay (*K*_decay_) and the mRNA concentration (*C*), leading to the following equation:

$$\mathrm{dC}/\mathrm{dt} = -{K}_{decay} C$$

Suppose *C*_t_, *C*_0_ respectively represents mRNA quantity at time *t* and time 0. The equation can be converted to:

$$ln({C}_{t}/{C}_{0}) = -{K}_{\mathrm{decay}} t$$

To calculate the mRNA half-life (*t*_1/2_), when half mRNA is decayed (that is, *C*_t_/*C*_0_ = 1/2), the final half-life equation is:

$$ln(1/2) =-{K}_{decay} {t}_{1/2}$$

$${t}_{1/2} = ln2/{K}_{decay}$$

### Analysis of ribo-seq data

For ribosome profiling analysis, the trimmed reads ≥ 20 nt in length were selected and mapped to the reference genome (TAIR10) [47] with HISAT2 (v2.1.0) [48]. FPKM was estimated for each gene by StringTie (v1.3.5) and R package (Ballgown) [49, 50]. Genes with FPKM > 1 in ribo-seq and FPKM > 1 in mRNA-seq were collected for the subsequent translation efficiency calculation. Translation efficiency (TE) was calculated comparing FPKM values of ribosome-bound fragments with mRNA FPKM values for the coding sequence (excluding UTRs) as the following equation: TE = FPKM_ribo-seq_/FPKM_RNA-seq_.

### Analysis of CLIP data

Transcripts of ECT2 targets and m^6^A modified sites were derived from published data. The ECT2 CLIP-seq data (GSE108119) was downloaded from NCBI GEO dataset and processed as reported method [14], and the m^6^A-seq data of WT (GSA: CA003050) was obtained from NGDC dataset. Bioinformatic analysis of m^6^A-seq data followed the steps as reported [18]. The transcripts with ECT2 CLIP targets are termed as ECT2 target genes, and those overlapped with m^6^A sites are termed as ECT2 & m^6^A targets. Transcripts without ECT2 CLIP targets or m^6^A sites are regarded as Non-targets. The PAB2- and PAB4-CLIP-seq data (GSE110342) were downloaded from the NCBI GEO and processed as previously reported [38]. The aligned reads were extended to 50 bp to identify significant PAB2 or PAB4 binding sites based on IP enrichment criteria (IP/input) ≥ 1 and *P*-value < 0.05 with MACS algorithm [52, 53]. The overlapped transcripts between ECT2 targets and PAB2 or PAB4 targets are termed as ECT2 & PAB2 common targets and ECT2 & PAB4 common targets, respectively. The remaining unbound genes are termed as Non-targets.

### Analysis of A-seq2 data

The A-seq2 data analysis was followed as previously described [31, 46]. For statistical analysis of PAC shift events, genes with at least two PACs were selected for analysis of APA shift. For analysis of genes with shifted PACs, the regions between the most proximal and most distal PACs were divided into two equal parts, and we pooled the TPM of each individual PAC in the 5′ half and in the 3′ half. PACs with TPM > 0 in both parts were used to calculate the genes with shifted PACs. Fisher’s exact test was used to identify the PAC shifted genes with *P*-value < 0.05. The length of 3′ UTR was defined as the distance from each PAC location to the stop codon (the sum of 3′ UTR length) multiplied by its expression level (TPM value) and then divided by the total expression level [54]. Two-sided *t* test was used to compare the relative abundance of 3′ UTR between *ect2/3/4* and WT samples.

## Supplementary Information

**Additional file 1: Supplementary Fig. S1-S18. Fig. S1.** BiFC assay showing the physical associations among ECT2, ECT3, and ECT4 in Nicotiana benthamiana leaf cells. **Fig. S2.** Characterization of the *ect2/3/4* mutant. **Fig. S3.** Phenotypic and statistical analysis of ABA sensitivity among WT, *ect2-1*, *ect3-2*, and *ect4-1*. **Fig. S4.** ECT2 depends on its m^6^A-binding function to play a core regulatory role in ECT2/ECT3/ECT4-mediated ABA response. **Fig. S5.** Correlation analysis of mRNA-seq between two biological replicates in WT and *ect2/3/4* mutant. **Fig. S6.** Reducing mRNA half-lives of ABA-related transcripts by silencing ECT2/ECT3/ECT4. **Fig. S7.** ECT2/ECT3/ECT4 enhance their targeted m^6^A-modified mRNA stabilization. **Fig. S8.** Confocal microscopy showing the cytoplasmic subcellular localization of ECT2 in *ECT2:ECT2-eGFP/ect2-1* transgenic *Arabidopsis* root tips. **Fig. S9.** Distribution and correlation analysis of A-seq2 profiling results. **Fig. S10.** ECT2/ECT3/ECT4 have no function in APA. **Fig. S11.** ECT2/ECT3/ECT4 have no function in translation. **Fig. S12.** ECT2 interacts with PAB proteins. **Fig. S13.** PAB2 and PAB4 promote mRNA stability. **Fig. S14.** ECT2 interacts with PAB4 to promote mRNA stability. **Fig. S15.** GO enrichment analysis of differential expressed genes in *ect2/3/4* mutant compared to WT. **Fig. S16.** ECT2 localizes in the cytoplasm under Mock and ABA treatment. **Fig. S17.** *DWA1*, *DWA2*, *SDIRIP1*, and *CPN20* transcripts containing m^6^A under ABA treatment. **Fig. S18.** PAB2 binds to *DWA1*, *DWA2*, *SDIRIP1*, and *CPN20* transcripts under Mock and ABA treatment. **Fig. S19.** The mRNA lifetime of negative control *AT2G07689* in 7-d-old WT and *ect2/3/4* seedlings. **Fig. S20.** The generation of *Crispr ABI5/ect2/3/4* mutants by CRISPR/Cas9 genome editing. **Fig. S21.** Statistical analysis of germination and of cotyledon greening rates in WT, *ect2/3/4*, *abi5-10*, and *Crispr ABI5/ect2/3/4* plants under Mock.

**Additional file 2: Table S1.** Statistical analysis of mRNA lifetime of ECT2 targets in WT and *ect2/3/4*.

**Additional file 3: Table S2.** Statistical analysis of ribo-seq among WT, *ect2-1*, and *ect2/3/4*.

**Additional file 4: Table S3.** Statistical analysis of ECT2 interacting proteins.

**Additional file 5: Table S4.** Differentially expressed genes between WT and *ect2/3/4*.

**Additional file 6: Table S5.** List of primers and oligonucleotides used in this study.

**Additional file 7.** Uncropped images for the blots in Figure 1, Figure 4 and Figure 6.

**Additional file 8.** Review history.

## Data Availability

The raw sequencing data of mRNA-Seq, ribo-Seq, mRNA lifetime-seq, and A-seq2 reported in this paper have been deposited in the Genome Sequence Archive in National Genomics Data Center, Beijing Institute of Genomics (BIG), Chinese Academy of Sciences (CRA005149), which is publicly accessible at https://bigd.big.ac.cn/gsa [55]. The published sequencing data related with PAB proteins can be downloaded from the NCBI database (GSE110342) [56]. All the other datasets supporting the conclusions in this study are included in the article and the Additional files.
