# Supplementary material for: m6A readers ECT2/ECT3/ECT4 enhance mRNA stability through direct recruitment of the poly(A) binding proteins in Arabidopsis
Source: Genome Biol. 2023 Apr 30;24:103. doi: 10.1186/s13059-023-02947-4 (PMC10150487; doi:10.1186/s13059-023-02947-4)
Supplement: Supplementary file 1 — Additional file 1: Supplementary Fig. S1-S18. Fig. S1. BiFC assay showing the physical associations among ECT2, ECT3, and ECT4 in Nicotiana benthamiana leaf cells. Fig. S2. Characterization of the ect2/3/4 mutant. Fig. S3. Phenotypic and statistical analysis of ABA sensitivity among WT, ect2-1, ect3-2, and ect4-1. Fig. S4. ECT2 depends on its m6A-binding function to play a core regulatory role in ECT2/ECT3/ECT4-mediated ABA response. Fig. S5. Correlation analysis of mRNA-seq between two biological replicates in WT and ect2/3/4 mutant. Fig. S6. Reducing mRNA half-lives of ABA-related transcripts by silencing ECT2/ECT3/ECT4. Fig. S7. ECT2/ECT3/ECT4 enhance their targeted m6A-modified mRNA stabilization. Fig. S8. Confocal microscopy showing the cytoplasmic subcellular localization of ECT2 in ECT2:ECT2-eGFP/ect2-1 transgenic Arabidopsis root tips. Fig. S9. Distribution and correlation analysis of A-seq2 profiling results. Fig. S10. ECT2/ECT3/ECT4 have no function in APA. Fig. S11. ECT2/ECT3/ECT4 have no function in translation. Fig. S12. ECT2 interacts with PAB proteins. Fig. S13. PAB2 and PAB4 promote mRNA stability. Fig. S14. ECT2 interacts with PAB4 to promote mRNA stability. Fig. S15. GO enrichment analysis of differential expressed genes in ect2/3/4 mutant compared to WT. Fig. S16. ECT2 localizes in the cytoplasm under Mock and ABA treatment. Fig. S17. DWA1, DWA2, SDIRIP1, and CPN20 transcripts containing m6A under ABA treatment. Fig. S18. PAB2 binds to DWA1, DWA2, SDIRIP1, and CPN20 transcripts under Mock and ABA treatment. Fig. S19. The mRNA lifetime of negative control AT2G07689 in 7-d-old WT and ect2/3/4 seedlings. Fig. S20. The generation of Crispr ABI5/ect2/3/4 mutants by CRISPR/Cas9 genome editing. Fig. S21. Statistical analysis of germination and of cotyledon greening rates in WT, ect2/3/4, abi5-10, and Crispr ABI5/ect2/3/4 plants under Mock. [file 13059_2023_2947_MOESM1_ESM.docx]

**Supplementary information**

# m^6^A readers ECT2/ECT3/ECT4 enhance mRNA stability through direct recruitment of the poly(A) binding proteins in *Arabidopsis*

Peizhe Song^1,^ **^†^**, Lianhuan Wei^1,^ **^†^**, Zixin Chen^1,^ **^†^**, Zhihe Cai^1^, Qiang Lu^1^, Chunling Wang^1^, Enlin Tian^1^, Guifang Jia^1,2,★^

^1^Synthetic and Functional Biomolecules Center, Beijing National Laboratory for Molecular Sciences, Key Laboratory of Bioorganic Chemistry and Molecular Engineering of Ministry of Education, College of Chemistry and Molecular Engineering, Peking University, Beijing 100871, China

^2^Peking-Tsinghua Center for Life Sciences, Beijing 100871, China

**^†^**These authors contributed equally: Peizhe Song, Lianhuan Wei, Zixin Chen.

^★^Corresponding authors: guifangjia@pku.edu.cn (G.J)

**Supplemental Figures and Figure legends**

**
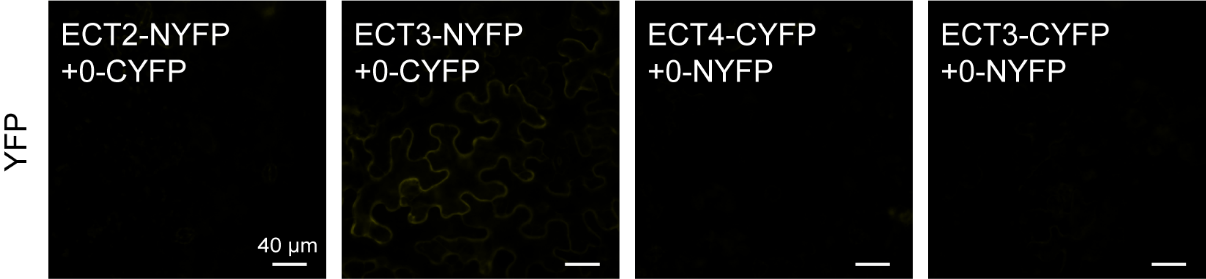
**

**Additional file 1: Fig. S1 BiFC assay showing the physical associations among ECT2, ECT3, and ECT4 in *Nicotiana benthamiana* leaf cells.** Scale bars = 40 μm.


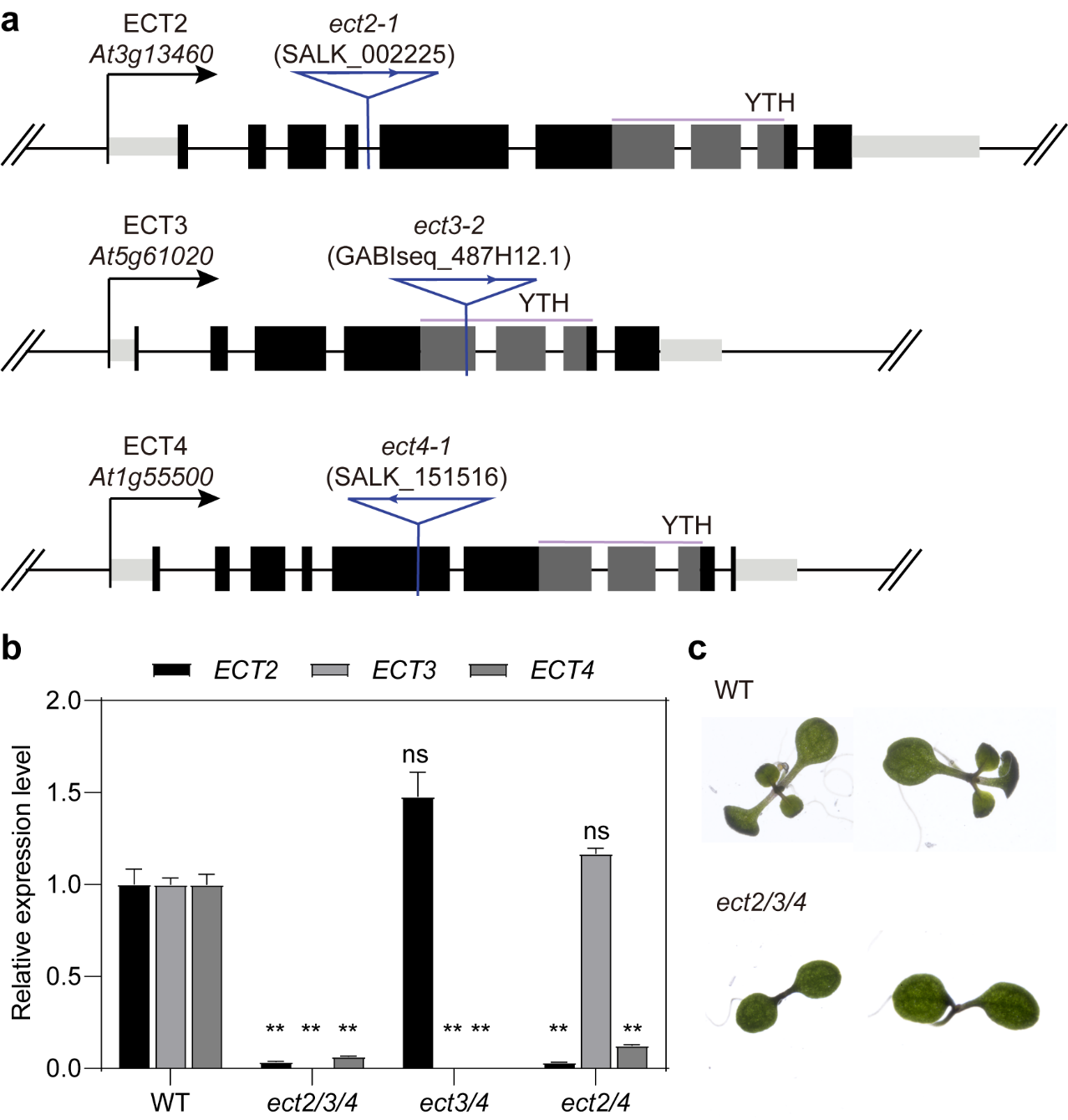


**Additional file 1: Fig. S2 Characterization of the *ect2/3/4* mutant. a** Schematic representation of the *ECT2*, *ECT3*, and *ECT4* loci and the T-DNA insertion line. Exons are depicted as boxes and introns as lines. **b** P RT-qPCR analysis of *ECT2, ECT3,* and *ECT4* expression levels in WT, *ect2/3/4*, *ect3/4*, and *ect2/4* mutants. *TUB8* is used as a reference gene. Data are presented as means ± SE, n = 2 biological replicates × 2 technical replicates. ***P* < 0.001 (two-sided *t*-test) **c** Phenotypes of 7-day-old seedlings of the indicated genotypes.


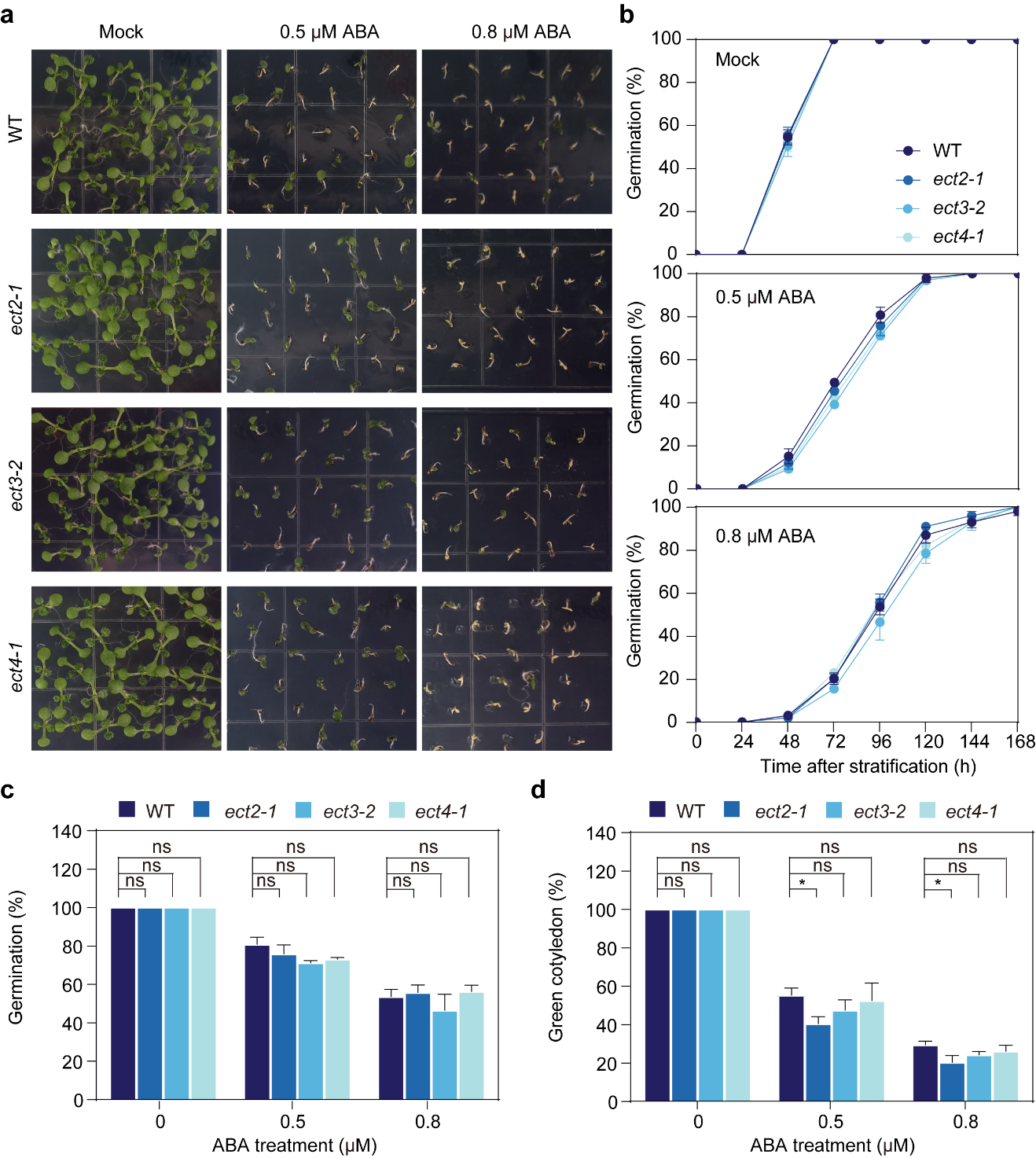


**Additional file 1: Fig. S3 Phenotypic and statistical analysis of ABA sensitivity among WT, *ect2-1*, *ect3-2*, and *ect4-1*.** Phenotypic analysis of the ABA response in WT, *ect2-1*, *ect3-2*, and *ect4-1*seeds grown on 1/2 MS-medium supplemented with 0 (Mock), 0.5, or 0.8 μM ABA under long-day conditions. Representative photographs were taken eight days after cold stratification. **b** Statistical analysis of germination rates in WT, *ect2-1,* *ect3-2* and *ect4-1* plants under ABA treatment. Radicle emergence was used as the morphological marker for germination. At least 30 seeds per genotype were measured in each replicate. Biological triplicates were averaged. Data are presented as the mean ± SE. **c-d** Statistical analysis of germination rates four days after imbibition (**c**) and of cotyledon greening rates eight days after imbibition (**d**) in WT, *ect2-1,* *ect3-2*, and *ect4-1* plants under ABA treatment. Data are presented as the mean ± SE; n = 3 biological replicates. **P* < 0.05 (two-sided *t-*test).


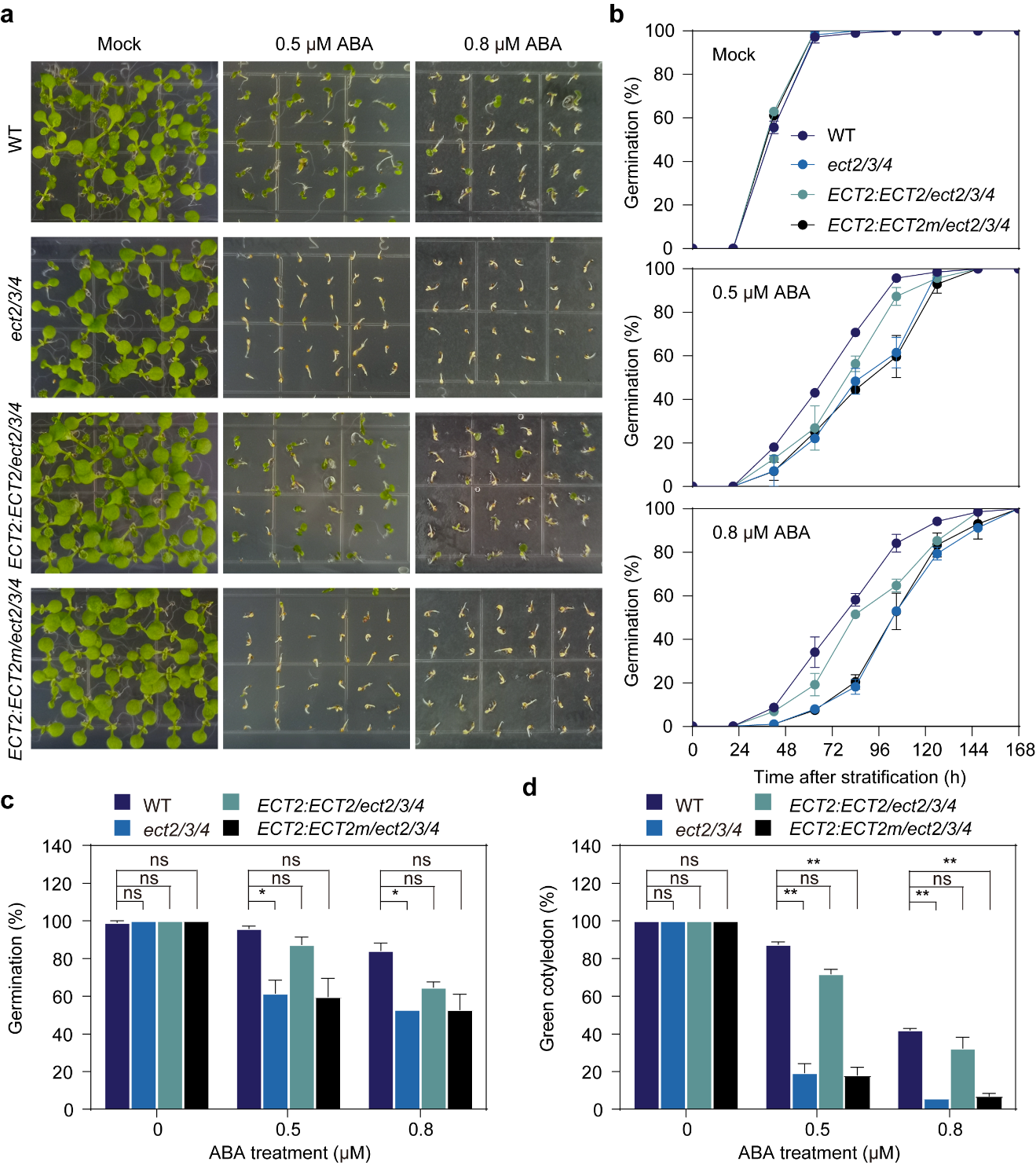


**Additional file 1: Fig. S4 ECT2 depends on its m^6^A-binding function to play a core regulatory role in ECT2/ECT3/ECT4-mediated ABA response. a** Phenotypic analysis of the ABA response in WT, *ect2/3/4*, *ECT2:ECT2/ect2/3/4*, and *ECT2:ECT2m/ect2/3/4* seeds grown on 1/2 MS-medium supplemented with 0 (Mock), 0.5, or 0.8 μM ABA under long-day conditions. Representative photographs were taken eight days after cold stratification. **b** Statistical analysis of germination rates in WT, *ect2/3/4*, *ECT2:ECT2/ect2/3/4*, and *ECT2:ECT2m/ect2/3/4* plants under ABA treatment. Radicle emergence was used as the morphological marker for germination. At least 30 seeds per genotype were measured in each replicate. Biological duplicates were averaged. Data are presented as the mean ± SE. **c-d** Statistical analysis of germination rates four days after imbibition **(c)** and of cotyledon greening rates eight days after imbibition **(d)** in WT, *ect2/3/4*, *ECT2:ECT2/ect2/3/4*, and *ECT2:ECT2m/ect2/3/4* plants under ABA treatment. Data are presented as the mean ± SE; n = 2 biological replicates. ***P* < 0.001, ****P* < 0.001, *****P* < 0.0001 (two-sided *t*-test).


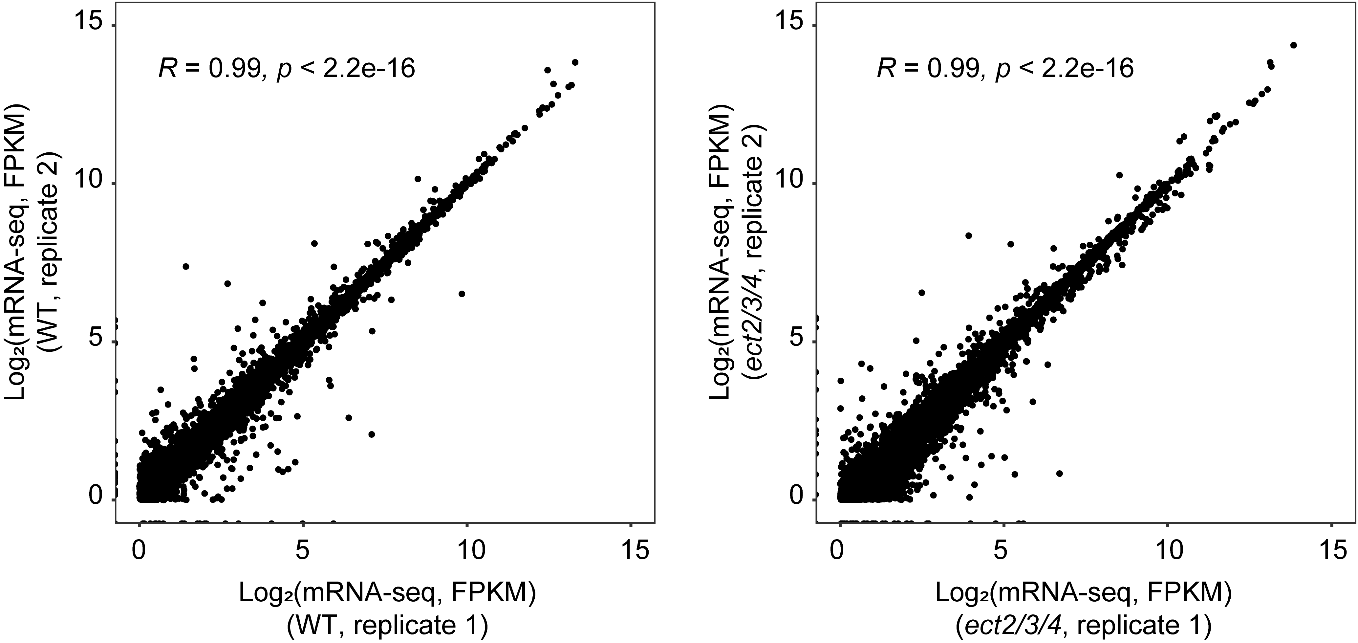


**Additional file 1: Fig. S5** **Correlation analysis of mRNA-seq between two biological replicates in WT and *ect2/3/4* mutant.**


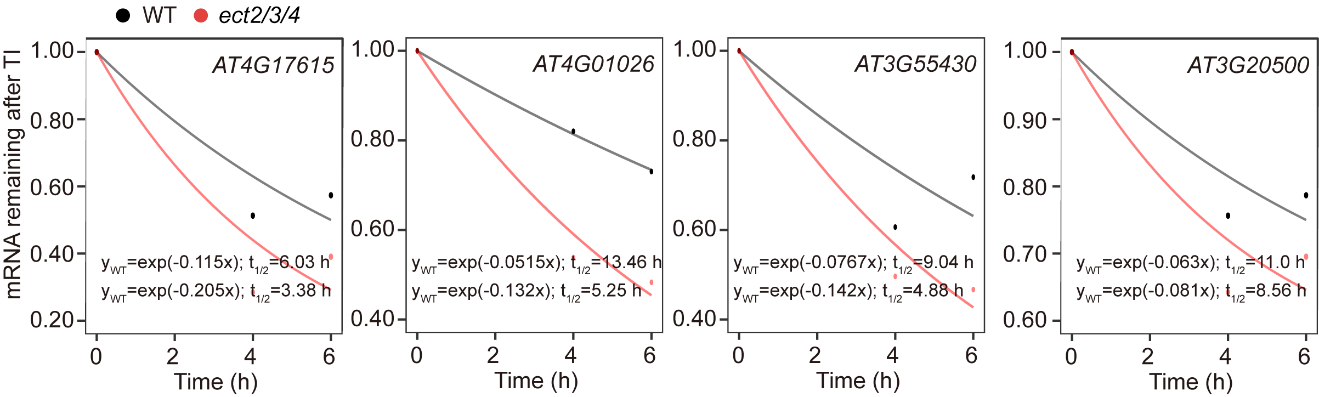


**Additional file 1: Fig. S6 Reducing mRNA half-life of ABA-related transcripts by silencing *ECT2/ECT3/ECT4*.**


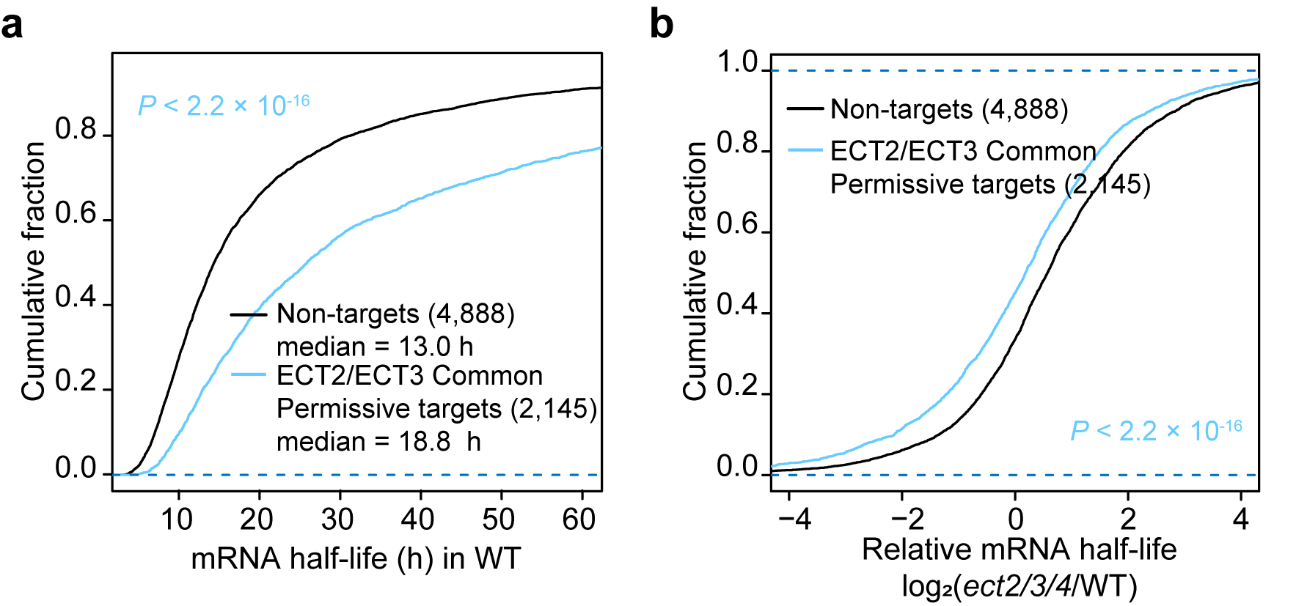


**Additional file 1: Fig. S7** **ECT2/ECT3/ECT4 enhance their targeted m^6^A-modified mRNA stabilization.** **a** Cumulative distribution of mRNA half-life in WT for Non-targets (black) and ECT2/ECT3 Common Permissive targets (blue).  **b** Cumulative distribution of relative mRNA half-life changes between *ect2/3/4* and WT for Non-targets (black) and ECT2/ECT3 Common Permissive targets (blue).


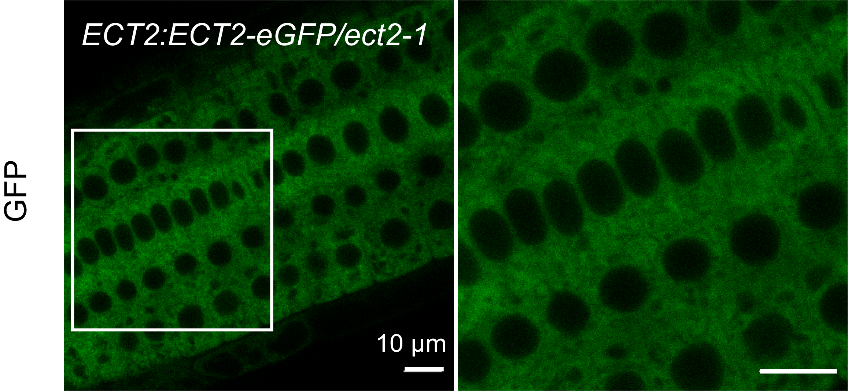


**Additional file 1: Fig. S8** **Confocal microscopy showing the cytoplasmic subcellular localization of ECT2 in *ECT2:ECT2-eGFP/ect2-1* transgenic *Arabidopsis* root tips.** Scale bars = 10 μm.


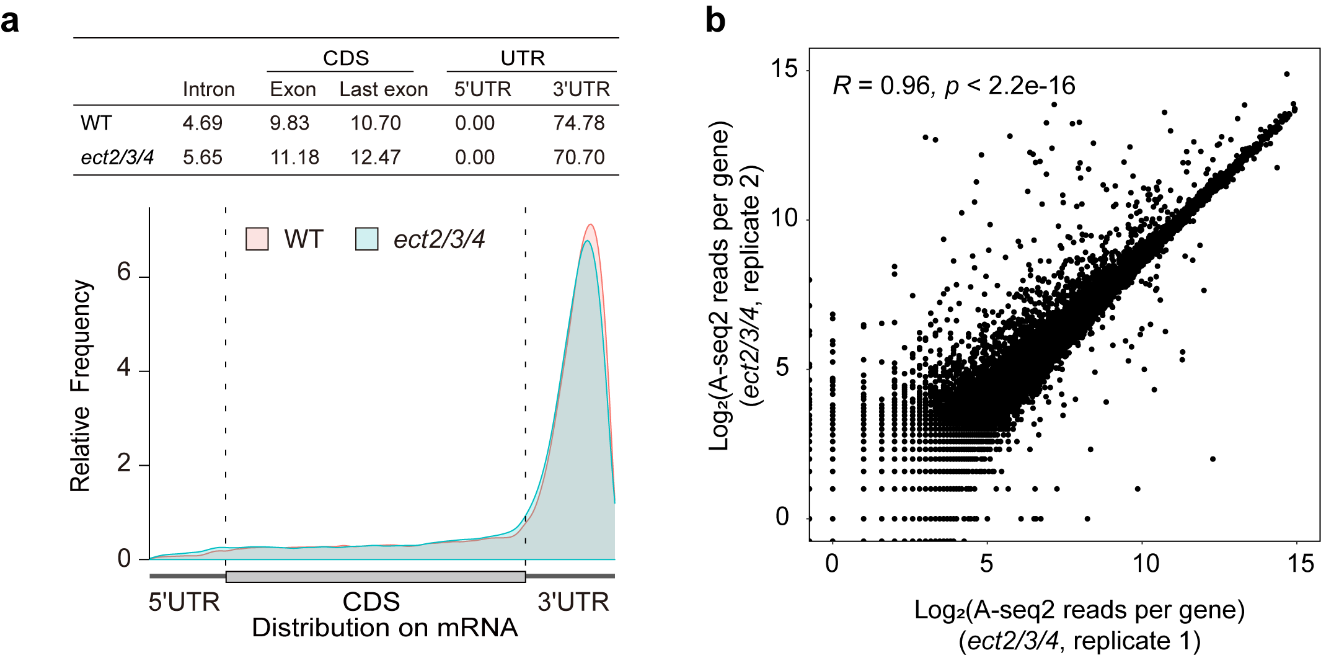


**Additional file 1: Fig. S9** **Distribution and correlation analysis of A-seq2 profiling results. a** Metagene proﬁle illustrating the region distribution of PACs across the indicated mRNA segments in WT and *ect2/3/4* mutant. **b** Correlation of A-seq2 sequencing reads per gene between two biological replicates in *ect2/3/4* mutant.


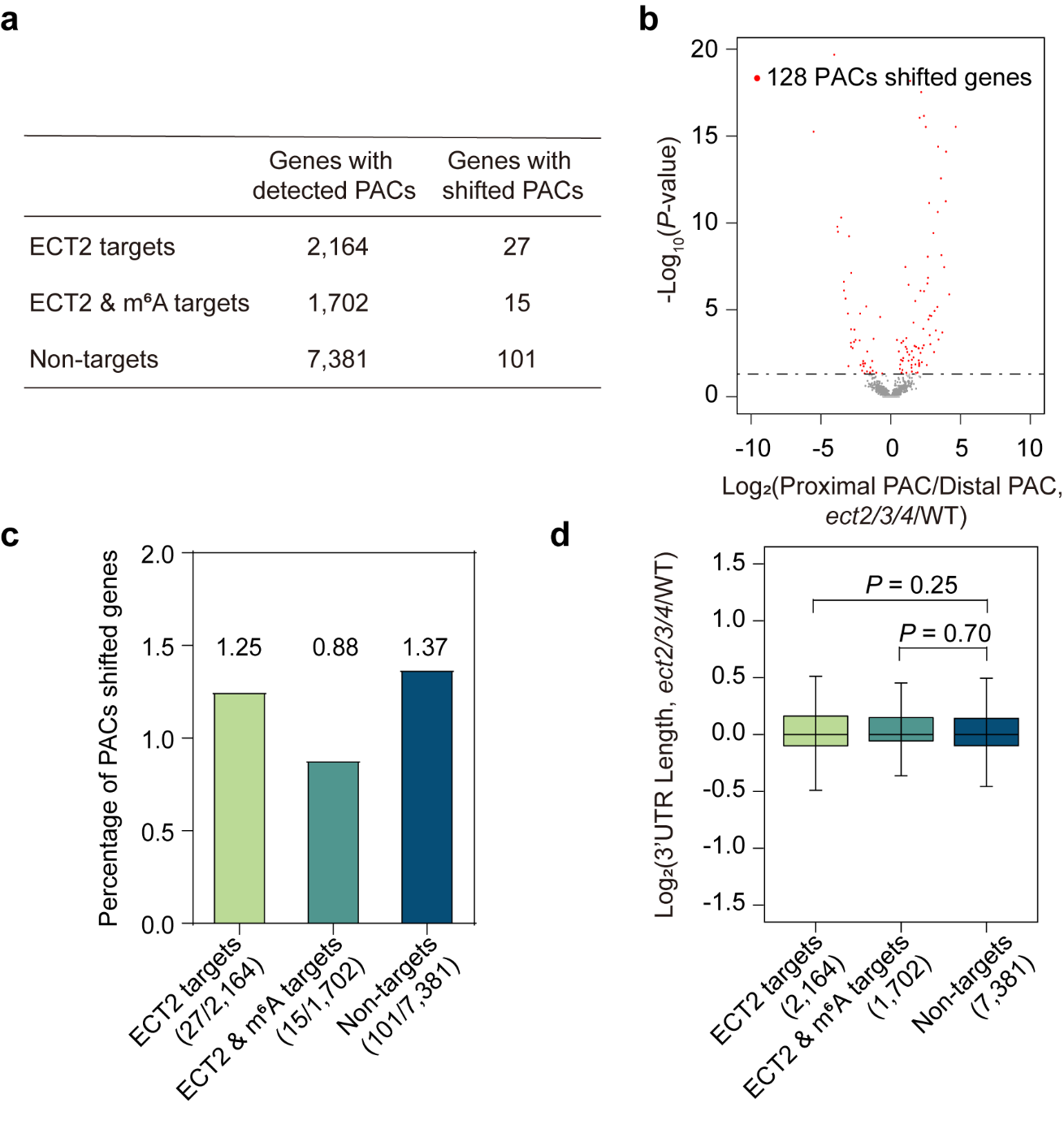


**Additional file 1: Fig. S10 ECT2/ECT3/ECT4 have no function in APA. a** Table showing genes with detected PACs and shifted PACs in ECT2 targets, ECT2 & m^6^A targets, and Non-targets. **b** Volcano plot depicting PAC-shifted genes (red points) in *ect2/3/4* compared to WT. PAC-shifted genes were classified using Fisher’s exact test (*p* < 0.05). **c** Percentage of PAC-shifted genes in *ect2/3/4* compared to WT for ECT2 targets, ECT2 & m^6^A targets, and Non-targets. **d** Relative 3' UTR average weighted length in *ect2/3/4* compared to WT for ECT2 targets, ECT2 & m^6^A targets, and Non-targets. *p*-values were calculated with Student’s *t*-test.

**
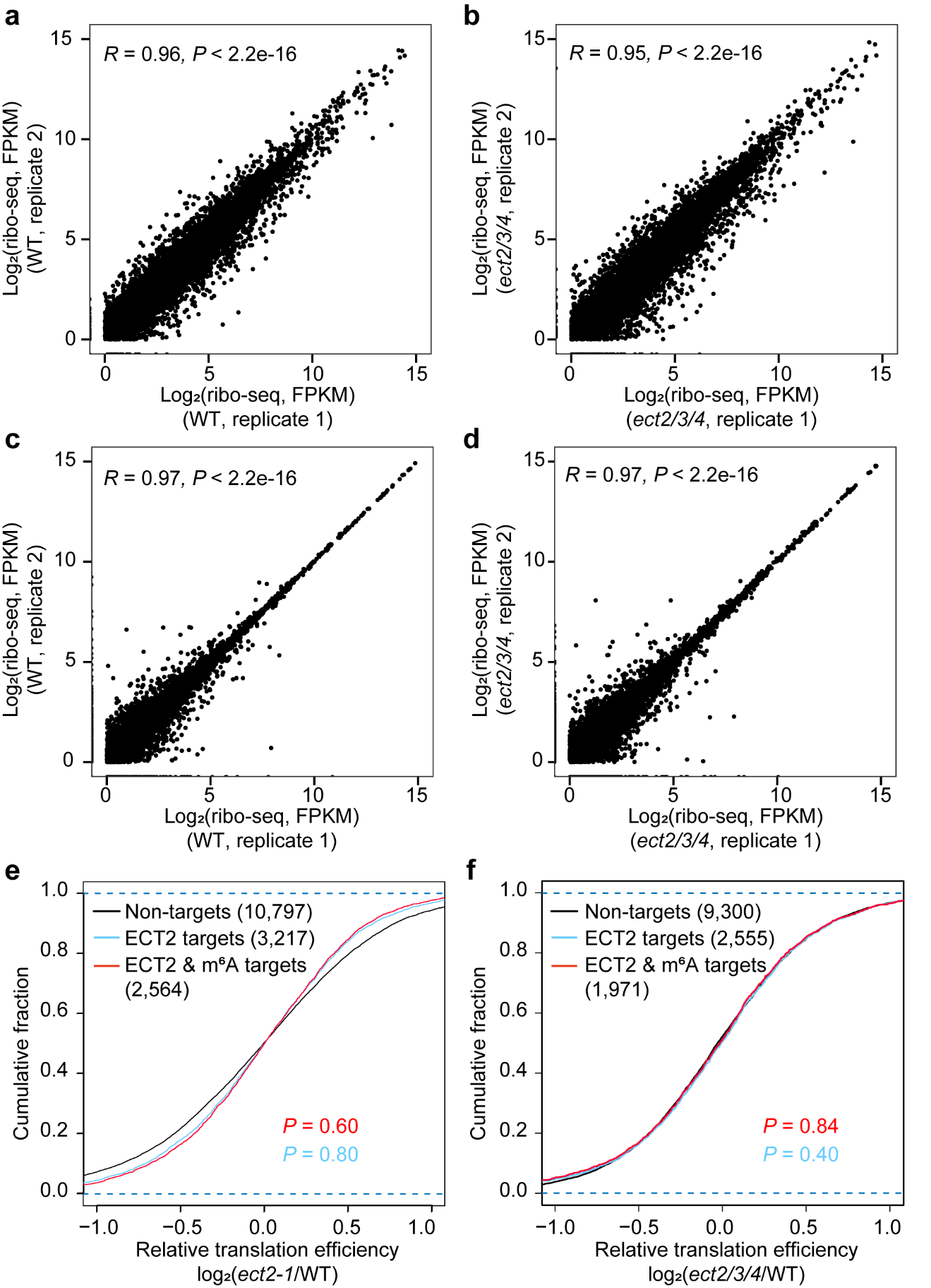
**

**Additional file 1: Fig. S11 ECT2/ECT3/ECT4 have no function in translation. a-b** Correlation analysis of ribo-seq between two biological replicates in WT and *ect2-1* mutant. **c-d** Correlation analysis of ribo-seq between two biological replicates in WT and *ect2/3/4* mutant. **e-f** Cumulative distribution of translation efficiency in *ect2-1* compared to WT **(e)** and *ect2/3/4* compared to WT **(f)** for Non-targets (black), ECT2 targets (blue), and ECT2 & m^6^A targets (red). *p* values were calculated using two-sided Mann-Whitney *U* test.


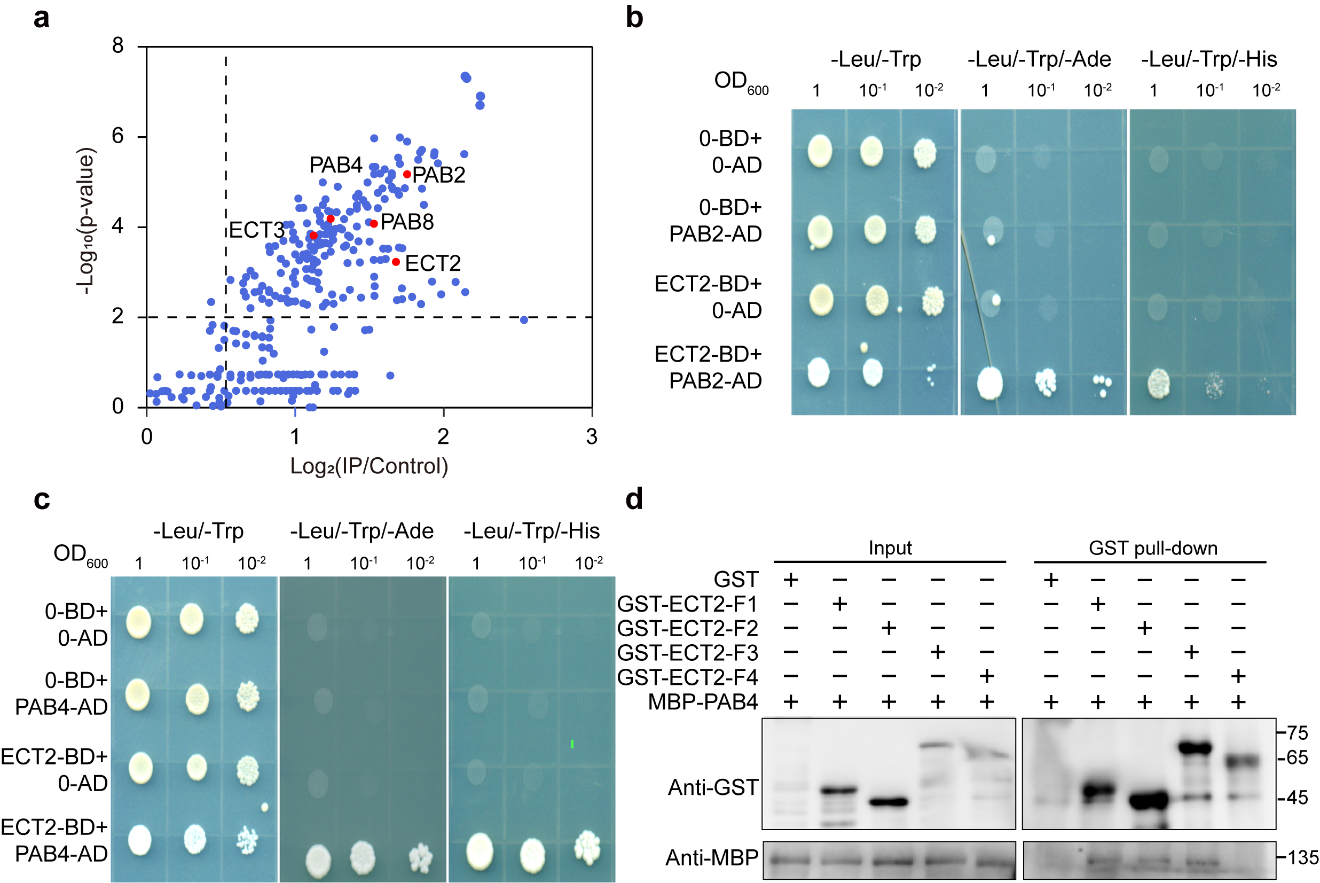


**Additional file 1: Fig. S12** **ECT2 interacts with PAB proteins. a** Scatterplot showing the proteins bound to endogenous *Arabidopsis* ECT2 without RNase treatment. The plot is based on the enrichment fold (IP/control) and *P* value. **b-c** Y2H assay showing the interaction of ECT2 with PAB2 (**b**) and PAB4 (**c**) in yeast cells. **d** Pull-down assay showing a direct interaction between PAB4 and PrLD of ECT2 *in vitro*. Purified MBP-PAB4 was incubated with GST-ECT2 fragments or GST alone, and pull-down assays were performed using GST magnetic beads, followed by immunoblot analysis with anti-GST and anti-MBP antibodies.


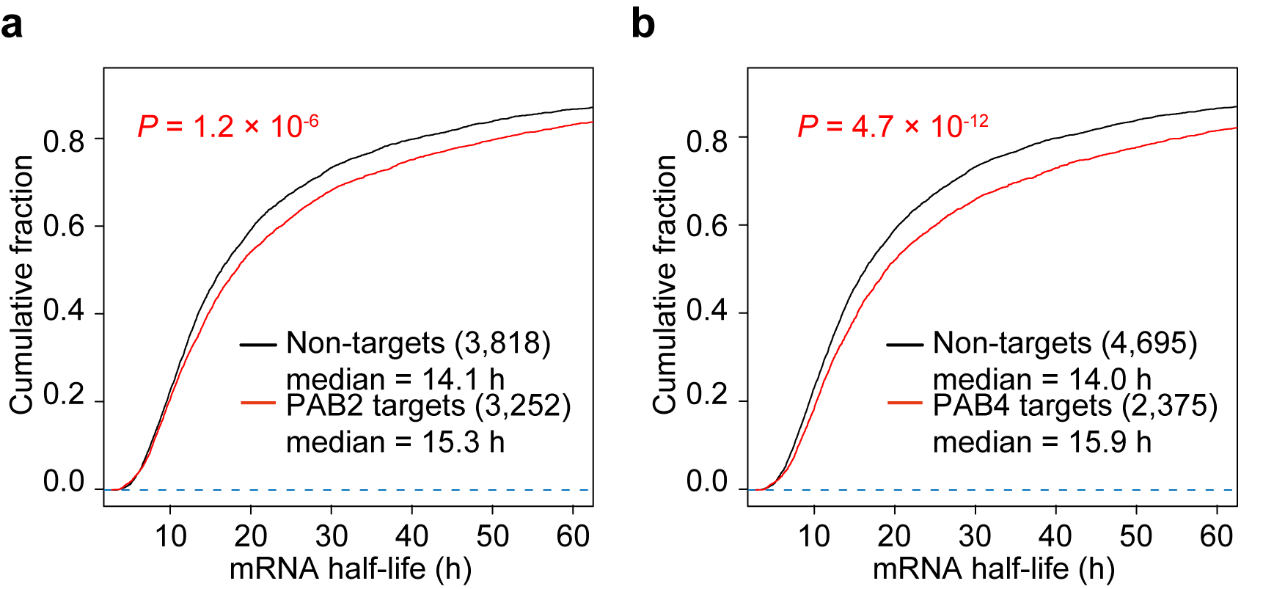


**Additional file 1: Fig. S13 PAB2 and PAB4 promote mRNA stability. a** Cumulative distribution of mRNA half-life in WT for Non-targets (black), PAB2 targets (red). **b** Cumulative distribution of mRNA half-life in WT for Non-targets (black), PAB4 targets (red). *p*-values were calculated using two-sided Mann-Whitney *U* test.


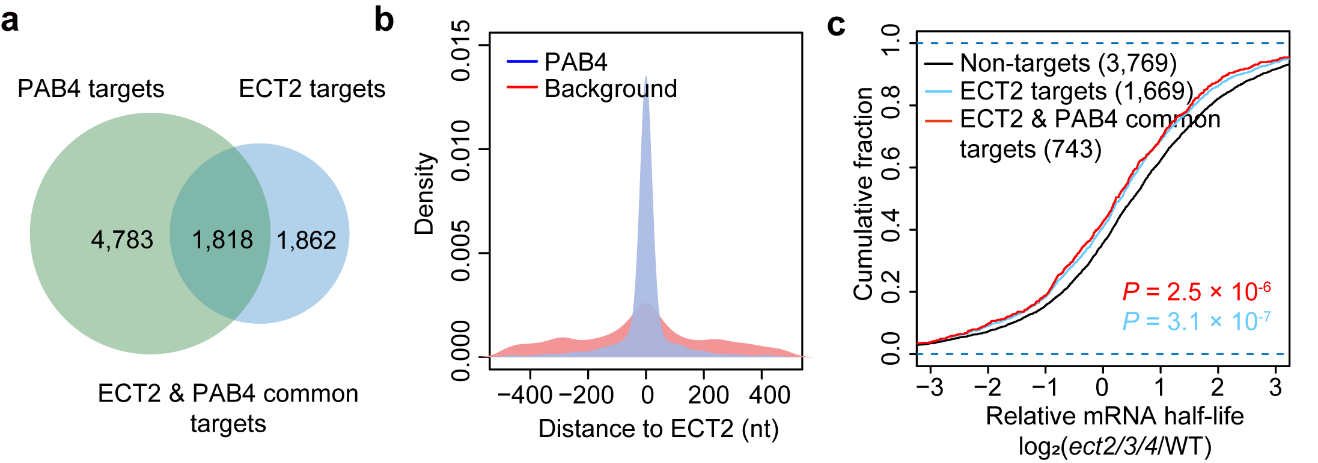


**Additional file 1: Fig. S14 ECT2 interacts with PAB4 to promote mRNA stability. a** Overlay of PAB4- and ECT2-binding targets in *Arabidopsis*. **b** The spatial distance distribution between the PAB4- and ECT2-bound regions. *P*-values were calculated using two-sided Mann-Whitney *U* test. **c** Cumulative distribution of relative mRNA half-life between *ect2/3/4* and WT for Non-targets (black), ECT2 targets (blue), and ECT2 & PAB2 common targets (red). *P*-values were calculated using two-sided Mann-Whitney *U* test.


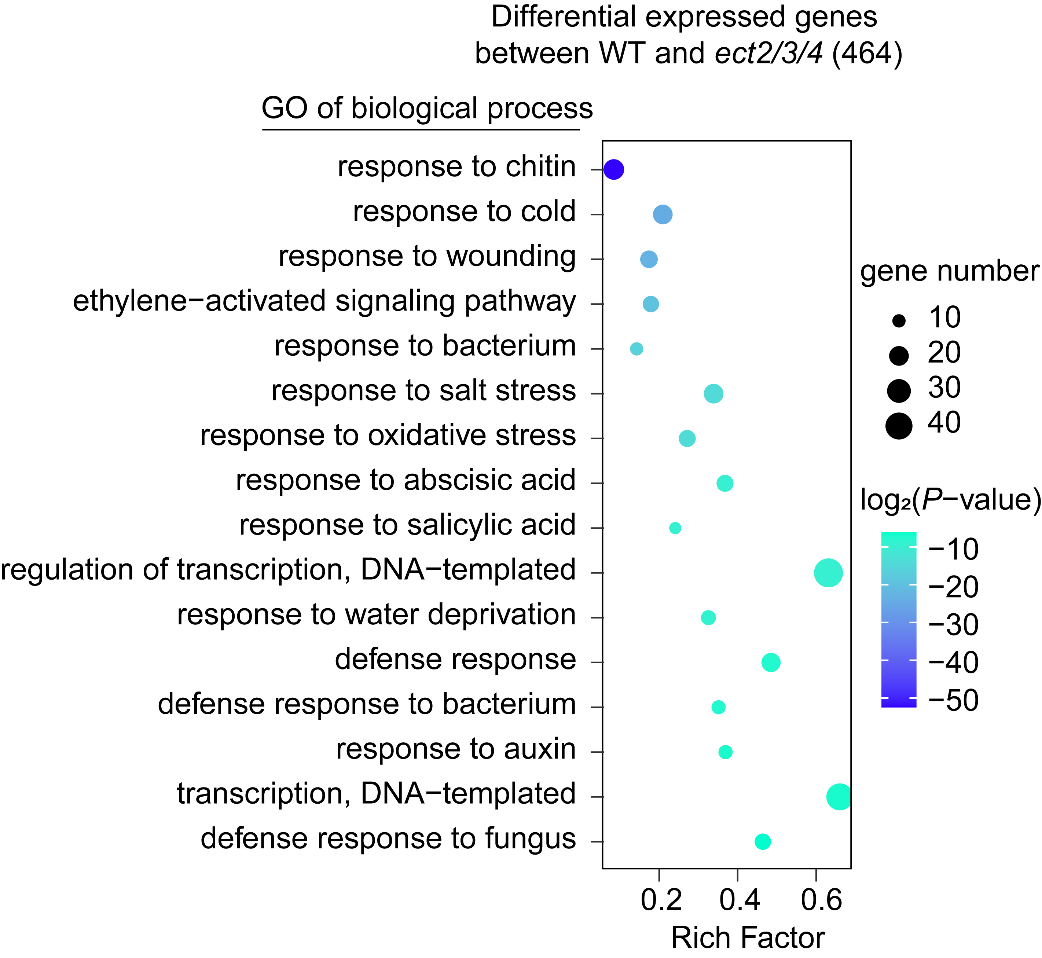


**Additional file 1: Fig. S15** **GO enrichment analysis of differential expressed genes in *ect2/3/4* mutant compared to WT.** The top 16 biological process terms are listed based on the gene counts.


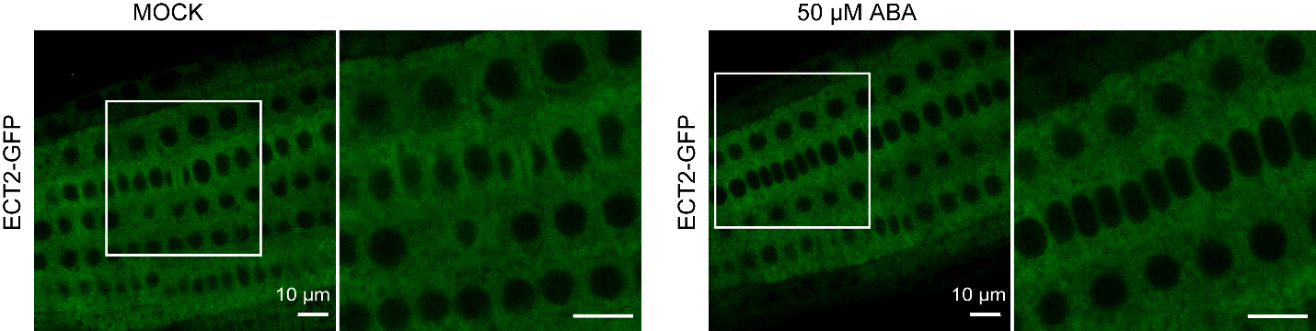


**Additional file 1: Fig. S16** **ECT2 localizes in the cytoplasm under Mock and ABA treatment.** Confocal images showing the cytoplasm localization of ECT2-GFP in root-tips of *ECT2:ECT2-eGFP/ect2-*1 under Mock and ABA treatment, Scale bar = 10 μm.


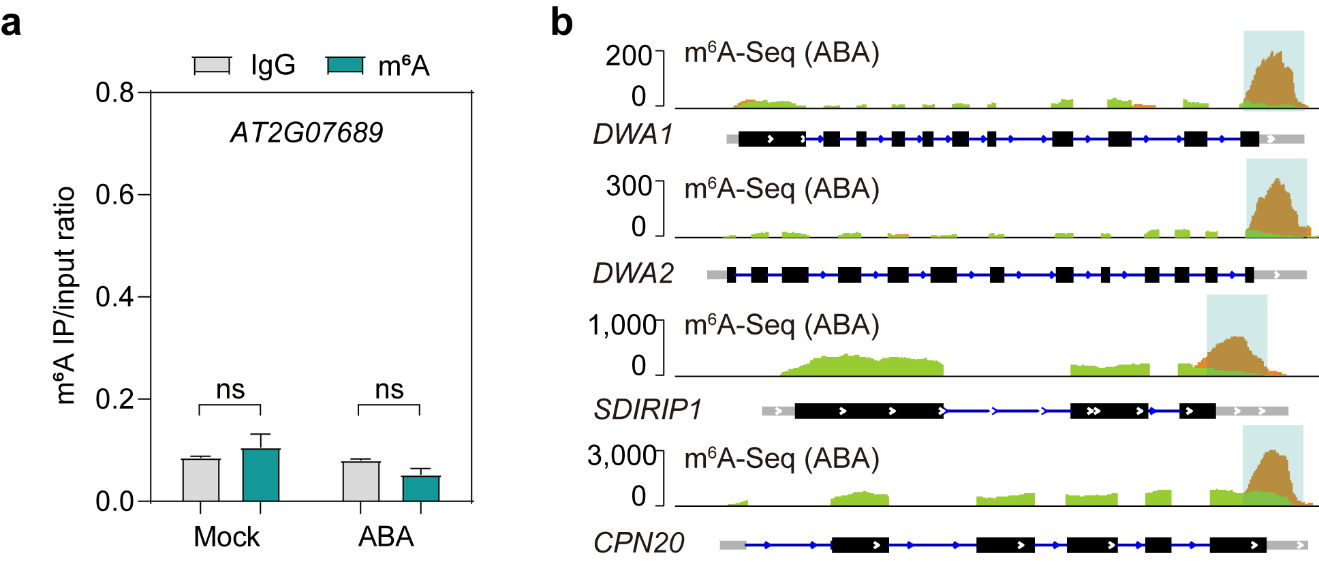


**Additional file 1: Fig.** **S17 *DWA1*, *DWA2*, *SDIRIP1*, and *CPN20* transcripts containing m^6^A under ABA treatment.** **a** m^6^A-IP-qPCR validation of the non-m^6^A peak in negative control under Mock and ABA treatment. **b** Integrative genomics viewer showing the m^6^A site on *DWA1*, *DWA2*, *SDIRIP1*, and *CPN20*. The sequencing data was from the published m^6^A sequencing results. The light blue box indicated the position of the m^6^A site.


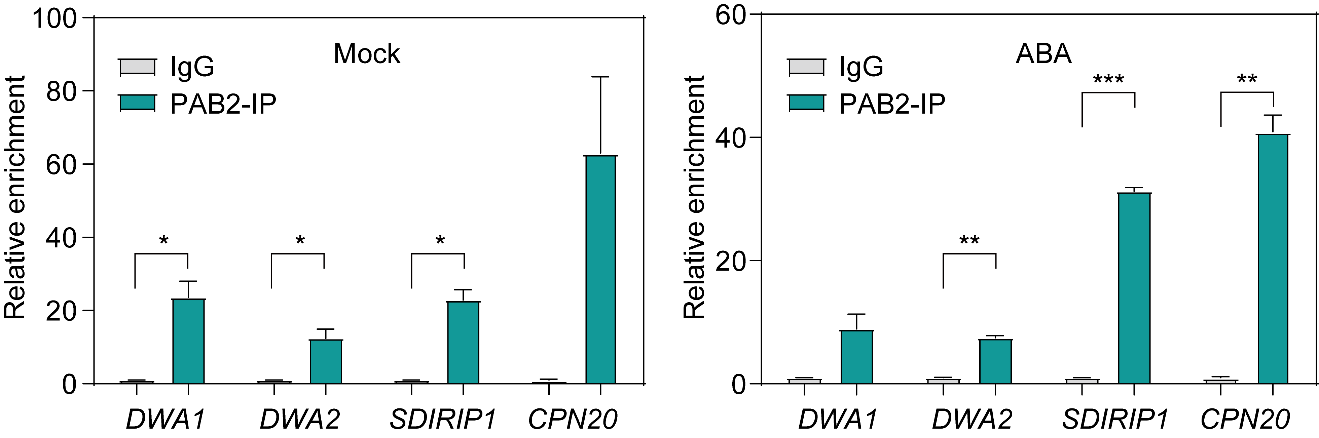


**Additional file 1: Fig. S18 PAB2 binds to *DWA1*, *DWA2*, *SDIRIP1*, and *CPN20* transcripts under Mock and ABA treatment.** FA-RIP-qPCR validation of the binding ability of PAB2 towards *DWA1*, *DWA2*, *SDIRIP1*, and *CPN20* in 12-d-old *PAB2:PAB2-Flag* seedlings under Mock and ABA treatment. Data are presented as means ± SE, n = 3 biological replicates × 2 technical replicates. **P* < 0.05, ***P* < 0.01, ****P* < 0.001 (two-sided *t-*test).

**
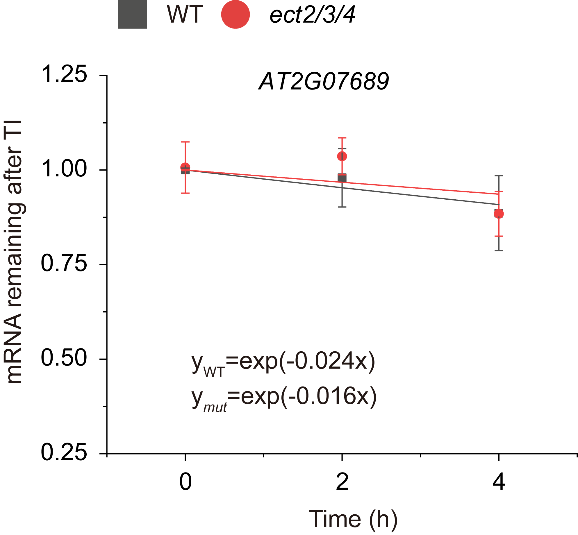
**

**Additional file 1: Fig. S19** **The mRNA lifetime of negative control *AT2G07689* in 7-d-old WT and *ect2/3/4* seedlings.** TI, transcription inhibition. *18S* was used as the internal control gene. Data are presented as means ± SE, n = 3 biological replicates × 2 technical replicates.

**
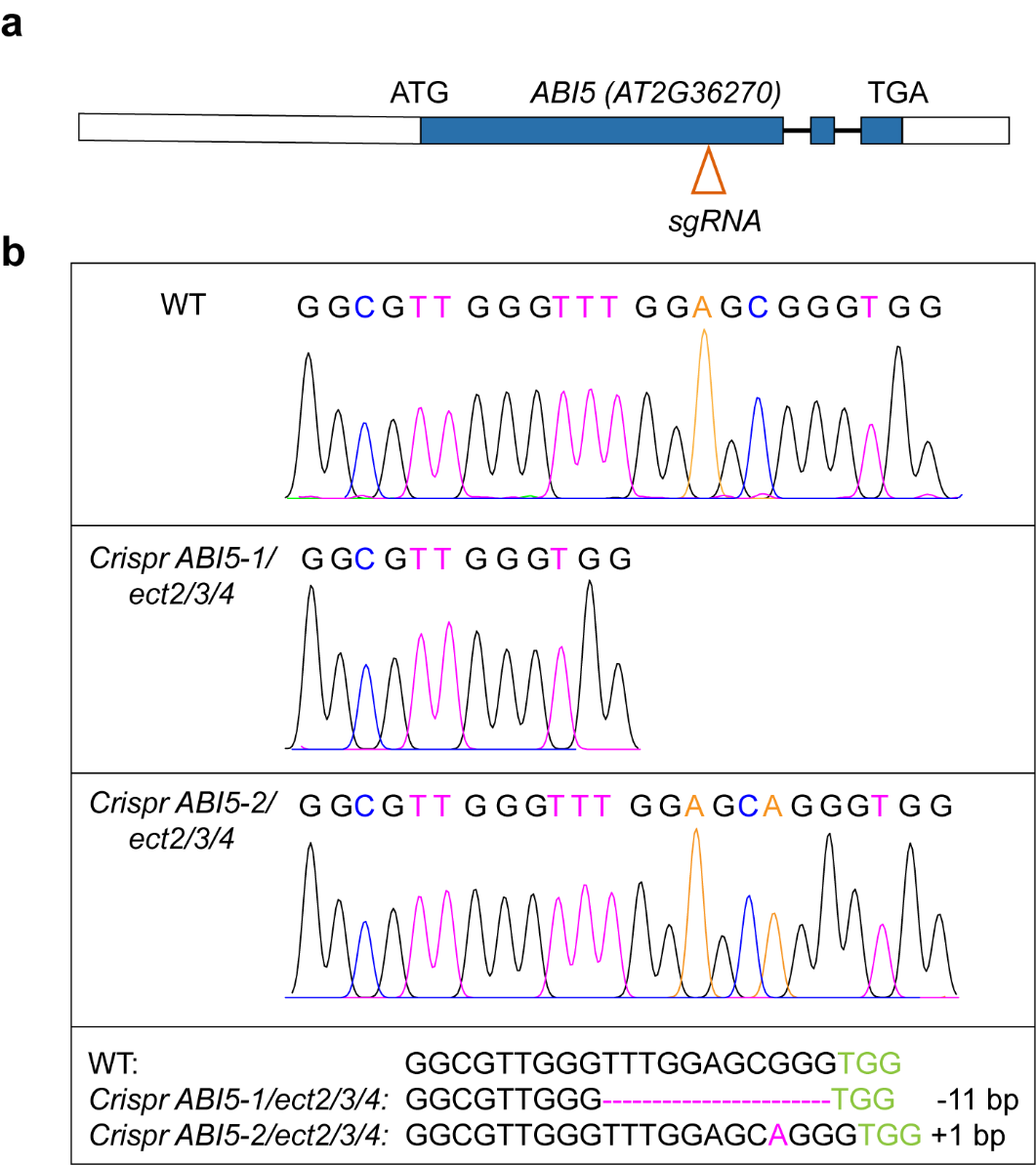
**

**Additional file 1: Fig. S20 The generation of *Crispr ABI5/ect2/3/4* mutants by CRISPR/Cas9 genome editing. a** Diagram showing the location of sgRNA-edited site in *ABI5* genome. Blue boxes: exons; Dark lines: introns; White boxes: 5' and 3' UTRs. Orange triangles: the editing site of sgRNA. **b** Sanger sequencing chromatograms showing the sgRNA-edited nucleotide sequences in two different mutant lines.

**
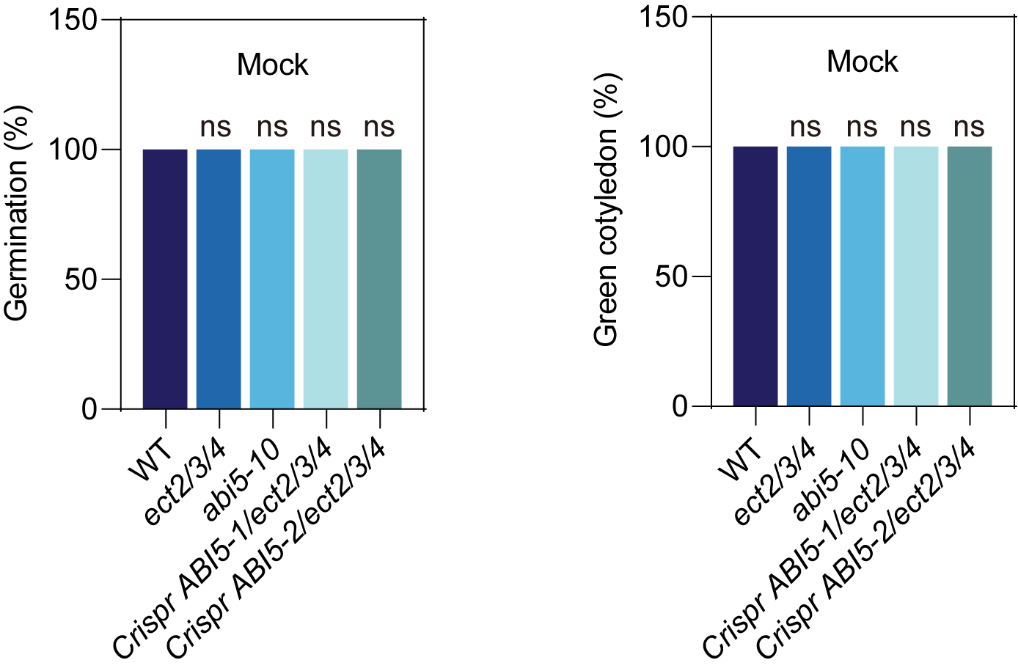
**

**Additional file 1: Fig. S21** Statistical analysis of germination and of cotyledon greening rates in WT, *ect2/3/4*, *abi5-10*, and *Crispr ABI5/ect2/3/4* plants under Mock. Germination and green cotyledon percentages were scored at 4 and 8 days after imbibition, respectively.
