# Supplementary material for: m6A readers ECT2/ECT3/ECT4 enhance mRNA stability through direct recruitment of the poly(A) binding proteins in Arabidopsis
Source: Genome Biol. 2023 Apr 30;24:103. doi: 10.1186/s13059-023-02947-4 (PMC10150487; doi:10.1186/s13059-023-02947-4)

Uncropped western blotting analysis

Uncropped western blotting and gel for Fig. 1


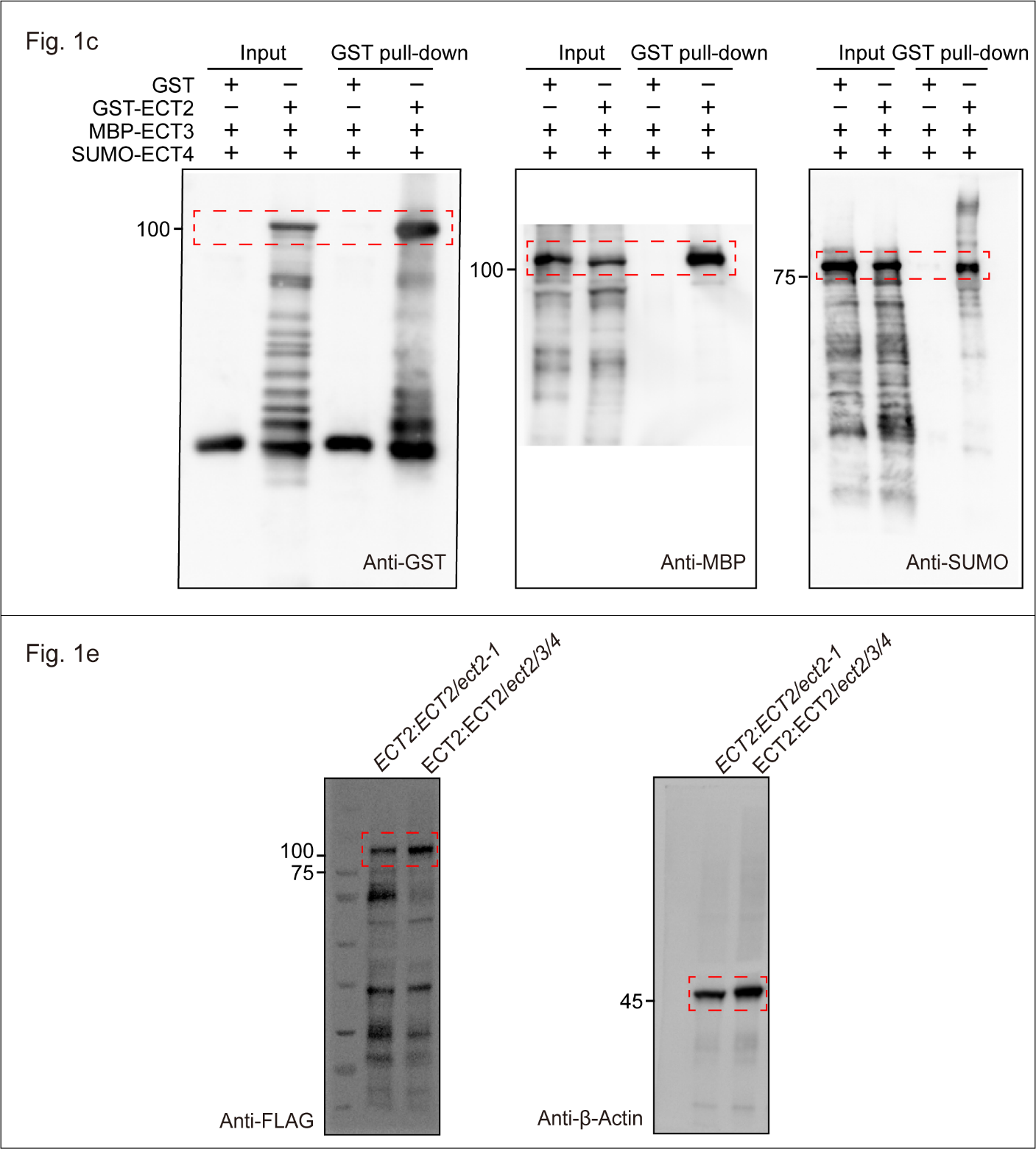


Uncropped western blotting and gel for Fig. 4


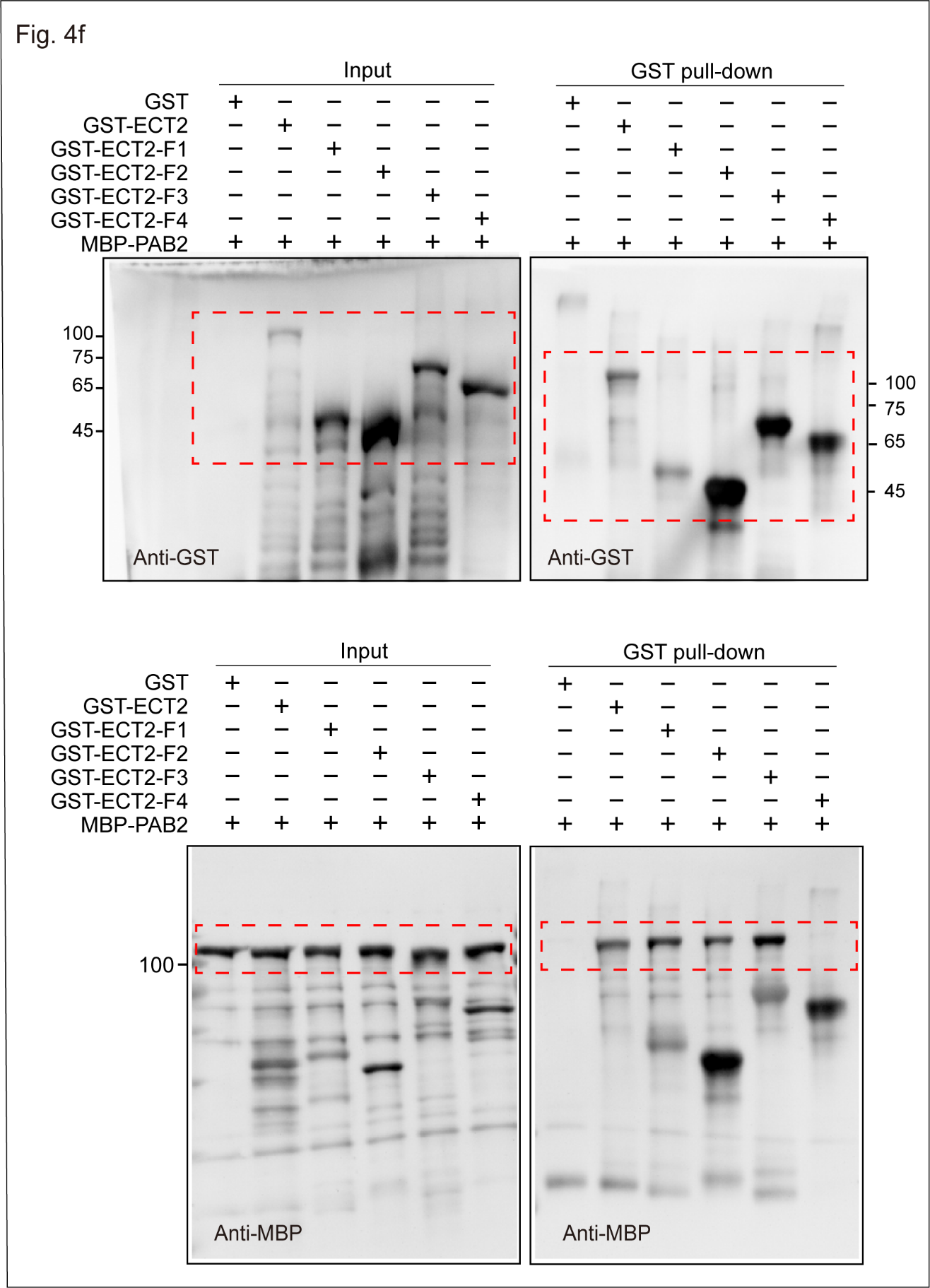


Uncropped western blotting and gel for Additional file 1: Fig. S12


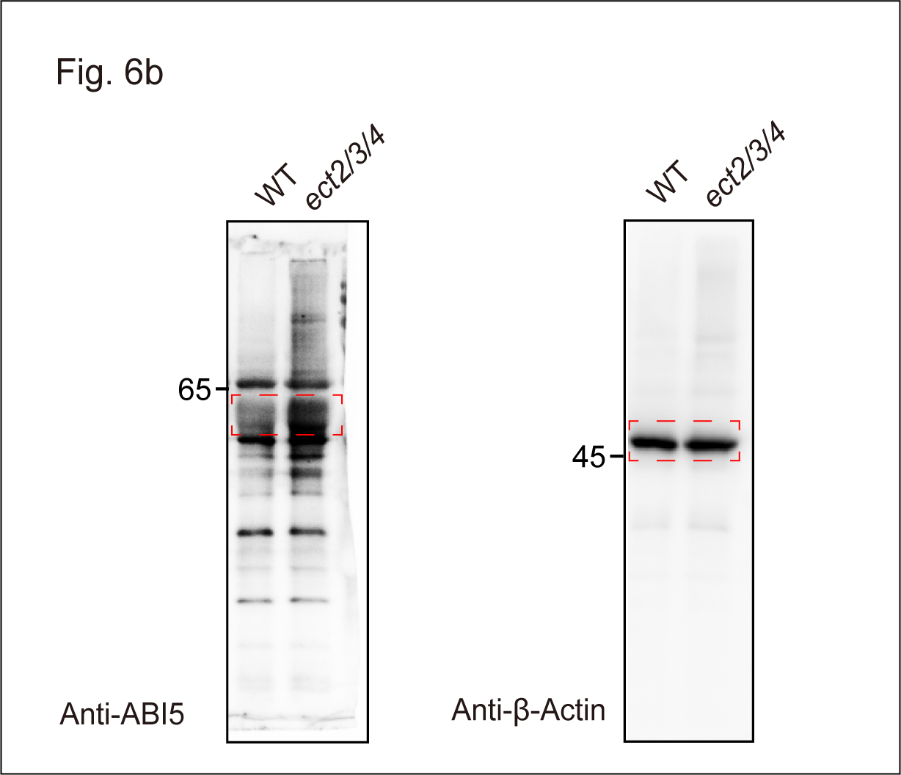


Uncropped western blotting and gel for Fig. 6


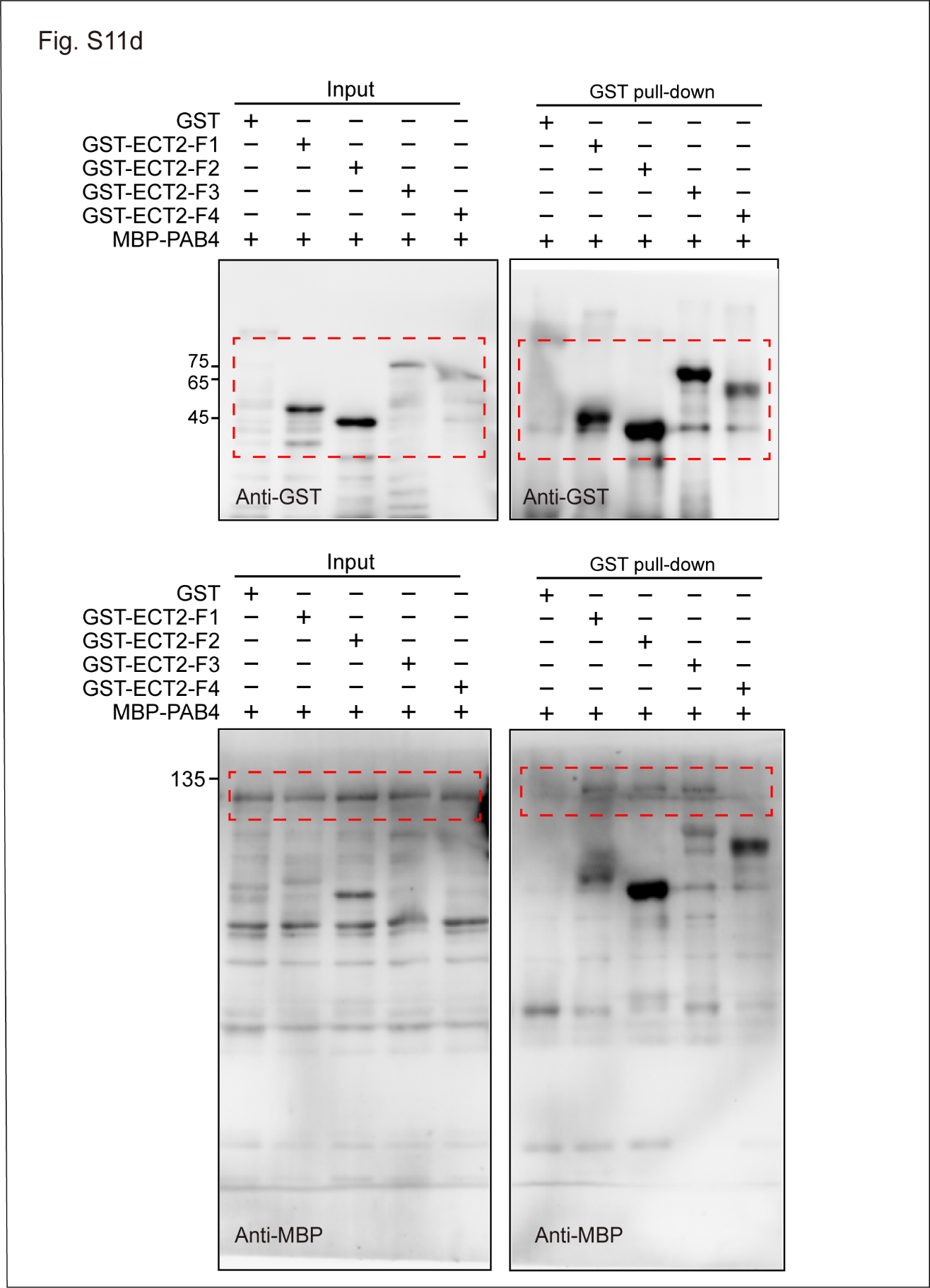

Supplement: Supplementary file 7 — Additional file 7. Uncropped images for the blots in Figure 1, Figure 4 and Figure 6. [file 13059_2023_2947_MOESM7_ESM.docx]
