# Supplementary material for: m6A readers ECT2/ECT3/ECT4 enhance mRNA stability through direct recruitment of the poly(A) binding proteins in Arabidopsis
Source: Genome Biol. 2023 Apr 30;24:103. doi: 10.1186/s13059-023-02947-4 (PMC10150487; doi:10.1186/s13059-023-02947-4)
Supplement: Supplementary file 8 — Additional file 8. Review history. [file 13059_2023_2947_MOESM8_ESM.docx]

Review History

**First round of review**

**Reviewer 1**

**Were you able to assess all statistics in the manuscript, including the appropriateness of statistical tests used?**

Yes, and I have assessed the statistics in my report.

**Comments to author:**

The authors revealed that the Arabidopsis m6A readers ECT2/ECT3/ECT4 promote mRNA stability via interacting with poly(A) binding proteins. They made serval interesting observations: 1) ECT2/ECT3/ECT4 directly interact with each other in the cytoplasm; 2) ECT2/ECT3/ECT4 have redundant functions in ABA response regulation; 3) ECT2/ECT3/ECT4 increase their target m6A-modified mRNA stabilization; 4) ECT2/ECT3/ECT4 do not affect alternative polyadenylation and translation; 5) ECT2 interacts with poly(A) binding proteins PAB2 and PAB4, and then coordinates with the stabilization of m6A-modified mRNAs.

Concerns:

Figure 6 better swaps with Figure 7.

m6A readers ECT2/ECT3/ECT4 form complex with PAB2/PAB4. Mammalian m6A readers YTHDC1 and YTHDF1-3 have separate functions in regulating mRNA splicing, export, stability and translation. What about ECT2, ECT3 or ECT4? Do they have separate function as well?

**Reviewer 2**

**Were you able to assess all statistics in the manuscript, including the appropriateness of statistical tests used?**

No, I do not feel adequately qualified to assess the statistics.

**Comments to author:**

In this manuscript, the authors employed multiple experimental and bioinformatical analysis to investigate the function of ECT2, ECT3 and ECT4 in ABA response. They showed that ECT2/ECT3/ECT4 may form a complex and redundantly function in cytoplasm. The ECT2 interacts with PAB2 and PAB4 and stabilized the m6A-modified mRNAs via binding to them. The complex formed by ECT and PAB proteins stabilized ABA response-related genes, and deficiency in ECT2/ECT3/ECT4 led to ABA hypersensitivity. The authors analyzed the molecular mechanism of ECT2/ECT3/ECT4 in mRNA stability. However, there are some issues remaining to be addressed by authors.

1. When measuring the germination rate of ect2-1, ect3-2, and ect4-1 single mutant seeds, the authors treated the mutants using 1uM ABA and no obvious differences in germination or cotyledon greening rates were observed. But when measuring the ABA sensitivity of double and triple mutants, the authors used different concentrations of ABA (0.5 uM and 0.8 uM) and revealed the predominant phenotype-related regulatory role of ECT2 in ECT2/ECT3/ECT4. So, if ECT2 play a key role, why there is no difference between ect2-1 and ect3-2 (or ect4-1)? When different concentrations of ABA (0.5, 1.0uM) were used, why were that?

2. The authors confirmed the triple knockout of ect2/3/4 mutant using RT-qPCR, but no proof was provided to confirm the expression level of the 3 genes in ect2/4 and ect3/4 mutants. The authors need to provide additional evidence.

3. Fig3 and 4i: I don't think the current accumulation figures are straightforward enough to convey the results. I am wondering if cumulative fraction line will be affected by different numbers between the non-targets and ECT2 targets, as non-targets may include more noises with extreme gene expression. Plots like volcano plot with statistical tests of individual genes (especially ABA pathway genes) would be preferred.

4. In Fig.4a, ECT3 was co-immunoprecipitated with ECT2. If the ECT2, ECT3 and ECT4 form a complex, why wasn't ECT4 co-immunoprecipitated? Although the Fig1 has proved that ECT2, ECT3 and ECT4 could interact with each other, but that is not a proof that ECT2, ECT3 and ECT4 form a complex containing three above proteins in vivo. Maybe the complex containing two proteins is enough to function, due to possible redundance of ECT2, ECT3 and ECT4. So the conclusion needs additional evidence to corroborate the 3-parts complex conclusion.

5. How is the m6A-seq performed? What is negative control in Fig 5 a/b? I do not find the description of m6A-seq in the methods. But clearly from the previous papers (PMID: 32313079; 34594034), extensive false positive m6A peaks can be found due to antibody bias with only 30% reproducibility. This is related to the m6A-IP-qPCR result, which is also based on pulled-down material by the antibody. Have the authors tried negative IgG control, which is recommended to eliminate the IgG antibody bias (PMID: 29941872)?

6. The figure 6 is a summary figure for this study. For that I would recommend putting this figure at the very last one as a conclusion. In the abstract, the authors stressed the function of CPSF30-L. Is it possible to put CPSF30-L also in the summary model?

Minor question:

1. In figure legend of Figure3, the 'a' and 'b' were put in wrong place. As the figure shows, 'a' represents ect2-1 compared to WT and 'b' represents ect2/3/4 compared to WT.

**Authors Response**

**Point-by-point responses to the reviewers’ comments:**

Referee #1:

The authors revealed that the Arabidopsis m6A readers ECT2/ECT3/ECT4 promote mRNA stability via interacting with poly(A) binding proteins. They made serval interesting observations: 1) ECT2/ECT3/ECT4 directly interact with each other in the cytoplasm; 2) ECT2/ECT3/ECT4 have redundant functions in ABA response regulation; 3) ECT2/ECT3/ECT4 increase their target m6A-modified mRNA stabilization; 4) ECT2/ECT3/ECT4 do not affect alternative polyadenylation and translation; 5) ECT2 interacts with poly(A) binding proteins PAB2 and PAB4, and then coordinates with the stabilization of m6A-modified mRNAs.

*Response: We thank this referee for the positive comments and thorough review of our work. The specific, point-by-point responses to the reviewer’s comments are addressed below.*

*1) Figure 6 better swaps with Figure 7.*

*Response: Thanks for the suggestions. We have swapped these figures in the revised manuscript (please see Fig. 8 in the revised manuscript).*

2) m6A readers ECT2/ECT3/ECT4 form complex with PAB2/PAB4. Mammalian m6A readers YTHDC1 and YTHDF1-3 have separate functions in regulating mRNA splicing, export, stability and translation. What about ECT2, ECT3 or ECT4? Do they have separate function as well?

*Response: Genetic redundancy is a common phenomenon in Arabidopsis (Trends Plant Sci 2006, 11(10), 492-8). Different from five members of mammalian YTH-domain family proteins (YTHDF1-3, YTHDC1-2), there are 13 YTH-domain family proteins in Arabidopsis (ECT1-11, CPSF30-L, and AT4G11970) (Ext.1 attached below) and comprehensive evolutionary analysis of these 13 YTH-domain family proteins proved that they are highly likely to be actual readers with redundant as well as specific functions (Plant Cell 2018, 33, 986–1005; Plant Cell 2018, 30, 952–967).*

*Ext. 1. Phylogenetic relationship of Arabidopsis YTH domain proteins (Plant Cell 2018, 30, 952–967).*

*Among these 13 YTH-domain family proteins, ECT2, ECT3, and ECT4 redundantly function in leaf growth, organogenesis, and ABA response (Development 2020, 147; Fig. 2 in the revised manuscript), and the binding targets between ECT2 and ECT3 are largely overlapped (Elife 2021, 10). Moreover, ECT2, ECT3, and ECT4 interact with each other (Fig. 1 in the revised manuscript), and disruption of ECT2, ECT3, and ECT4 affects their targeted m6A-modified mRNA stability and leads to more effective and significant reduction in transcript abundance than disruption of ECT2 alone (Fig. 3 in the revised manuscript). Additionally, we have shown that ECT2/ECT3/ECT4 don’t have function in APA regulation and translation (Additional file 1: Fig. S10, 11). Thus, these data confirmed that ECT2, ECT3, and ECT4 redundantly promote m6A-modified mRNA stability and regulate mRNA abundance, and they don’t have separate function in other stages of mRNA processing, which should be regulated by other Arabidopsis m6A readers.*

Referee #2:

In this manuscript, the authors employed multiple experimental and bioinformatical analysis to investigate the function of ECT2, ECT3 and ECT4 in ABA response. They showed that ECT2/ECT3/ECT4 may form a complex and redundantly function in cytoplasm. The ECT2 interacts with PAB2 and PAB4 and stabilized the m6A-modified mRNAs via binding to them. The complex formed by ECT and PAB proteins stabilized ABA response-related genes, and deficiency in ECT2/ECT3/ECT4 led to ABA hypersensitivity. The authors analyzed the molecular mechanism of ECT2/ECT3/ECT4 in mRNA stability. However, there are some issues remaining to be addressed by authors.

*Response: We thank this referee for the positive comments and thorough review of our work. The specific, point-by-point responses to the reviewer’s comments are addressed below.*

1) When measuring the germination rate of ect2-1, ect3-2, and ect4-1 single mutant seeds, the authors treated the mutants using 1uM ABA and no obvious differences in germination or cotyledon greening rates were observed. But when measuring the ABA sensitivity of double and triple mutants, the authors used different concentrations of ABA (0.5 uM and 0.8 uM) and revealed the predominant phenotype-related regulatory role of ECT2 in ECT2/ECT3/ECT4. So, if ECT2 play a key role, why there is no difference between ect2-1 and ect3-2 (or ect4-1)? When different concentrations of ABA (0.5, 1.0uM) were used, why were that? germination or cotyledon greening rates measurement of ect2-1, ect3-2, and ect4-1 single mutant seeds under different concentrations of ABA (0.5 uM and 0.8 uM)

*Response: Thanks for the suggestion. In the revision, we evaluated ABA sensitivity by comprehensive measurement of germination and cotyledon greening rates among WT, ect2-1, ect3-2, and ect4-1 under different ABA concentrations (0.5 µM and 0.8 µM). The statistical results showed that the germination rates of the mutant seeds were indistinguishable from the WT seeds under ABA (Fig. S3a-c in the revised manuscript). As reported that cotyledon greening is more strongly inhibited by ABA than germination (Plant Physiol 2014, 164, 1515-1526), indeed, we also observed that the cotyledon greening rates in ect2-1 were significantly reduced compared to WT in the presence of 0.5 µM and 0.8 µM ABA. The ect3-2 and ect4-1 mutant seeds also exhibited a slight (but not significant) cotyledon greening rate reduction compared to WT (Fig. S3d in the revised manuscript). The results suggested that ECT2, ECT3, and ECT4 redundantly regulate ABA response, in which ECT2 plays a predominant role in it. We have re-written the results in the revised manuscript.*

2) The authors confirmed the triple knockout of ect2/3/4 mutant using RT-qPCR, but no proof was provided to confirm the expression level of the 3 genes in ect2/4 and ect3/4 mutants. The authors need to provide additional evidence.

*Response: Thanks for the suggestion. We have performed RT-qPCR to detect the expression level of ECT2, ECT3, and ECT4 in ect2/4, ect3/4, and ect2/3/4 mutants, respectively (please see Fig. S2b in the revision). The results showed that compared with WT, the expression of ECT2 and ECT4 were not almost expressed in ect2/4 mutant, and the expression of ECT3 and ECT4 were almost completely reduced in ect3/4 mutant, confirming the double knockout of ect2/4 and ect3/4 mutants.*

*Moreover, we found that ECT2 expression in ect3/4 and ECT3 expression in ect2/4 mutant were compensatory increased, suggesting that ECT2, ECT3, and ECT4 redundantly function in biological process.*

3) Fig3 and 4i: I don't think the current accumulation figures are straightforward enough to convey the results. I am wondering if cumulative fraction line will be affected by different numbers between the non-targets and ECT2 targets, as non-targets may include more noises with extreme gene expression. Plots like volcano plot with statistical tests of individual genes (especially ABA pathway genes) would be preferred.

*Response: Thanks for the suggestion, in our opinion, most of the statistical evaluations rely on the cumulative plot. Even though either histogram or volcano plot seems to be more intuitive at the first look and needs less explanation, the main advantages of the cumulative plot are the fact that main and important key values and features like minimum, maximum, median, quantiles, percentiles, etc. can be directly read from the diagram, and cumulative plot can be defined for any kind of random variable (discrete, continuous, and mixed). What is more, cumulative plot has been widely utilized to illustrate changes for transcriptomic RNA stability (Nature 2014, 505(7481), 117-20; Nat Cell Biol 2018, 20(3), 285-295; Proc Natl Acad Sci U S A 2020, 117(48), 30400-30411). Thus, we think that the cumulative plot analysis in fig. 3 and fig. 4i can clearly and effectively exhibit the half-life difference between WT and ect2/3/4 mutant.*

*Additionally, we exhibited the mRNA decay rate of several representative ECT2 & m6A targets involved in ABA response using the sequencing data of mRNA stability (Additional file 1: Fig. S6), which could better illustrate the enhanced mRNA stability functions of ECT2/ECT3/ECT4 in ABA response.*

4) In Fig.4a, ECT3 was co-immunoprecipitated with ECT2. If the ECT2, ECT3 and ECT4 form a complex, why wasn't ECT4 co-immunoprecipitated?

*Response: Our ribo-seq database showed that the protein level of ECT4 is only eighth of ECT3 (Ext.2 attached below). Due to the differential protein level, some high-abundance ECT2-interacting proteins such as ECT3 are easily immunoprecipitated in the ECT2-IP assay, which may cause the missing of low-abundance ECT2-interacting proteins, like ECT4.*

*Additionally, the plant.MAP database showed that ECT2 can form the complex together with ECT4 (Cell 2020, 181(2), 460-474), in consistent with the physical interaction among ECT2, ECT3, and ECT4 (Fig.1 in the revised manuscript).*

*Ext. 2. Ribo-seq FPKM of ECT3 and ECT4 in WT.*

Although the Fig1 has proved that ECT2, ECT3 and ECT4 could interact with each other, but that is not a proof that ECT2, ECT3 and ECT4 form a complex containing three above proteins in vivo. Maybe the complex containing two proteins is enough to function, due to possible redundance of ECT2, ECT3 and ECT4. So the conclusion needs additional evidence to corroborate the 3-parts complex conclusion.

*Response: To further study the specific regulatory mechanism of ECT2/ECT3/ECT4 complex, in the revised manuscript we generated a new ECT2:ECT2-Flag/ect2/3/4 transgenic plant that endogenously expressed ECT2 in ect2/3/4 background. We conducted the FA-RIP assay with ECT2:ECT2-Flag/ect2/3/4 and previously generated ECT2:ECT2-Flag/ect2-1 transgenic plants, in which the protein expression level of ECT2 were similar in both plants (Fig. 1e in the revised manuscript). We observed that the immuoprecipitated RNA amount and m6A level bound by ECT2 were dramatically reduced in ECT2:ECT2-Flag/ect2/3/4 plant compared to ECT2:ECT2-Flag/ect2-1 (Fig. 1f in the revised manuscript), suggesting that ECT2 together with ECT3 and ECT4 form a complex to bind m6A-modified transcripts more effectively and efficiently than ECT2 alone.*

5) How is the m6A-seq performed? What is negative control in Fig 5 a/b? I do not find the description of m6A-seq in the methods.

*Response: We used the previously published m6A-Seq data to analyze the downstream targets in the manuscript; therefore, we have not descripted the m6A-Seq method. We have re-descripted the usage of the published m6A-Seq in the revised manuscript. And we added the negative control in Additional file 1: Fig. S17a.*

But clearly from the previous papers (PMID: 32313079; 34594034), extensive false positive m6A peaks can be found due to antibody bias with only 30% reproducibility. This is related to the m6A-IP-qPCR result, which is also based on pulled-down material by the antibody. Have the authors tried negative IgG control, which is recommended to eliminate the IgG antibody bias (PMID: 29941872)?

*Response: In the revision, we performed the m6A-IP-qPCR with negative IgG control, and the results showed that DWA1, DWA2, SDIPRIP1, and CPN20 transcripts were modified with m6A under either Mock or ABA treatment (Fig. 5b in the revised manuscript).*

6) The figure 6 is a summary figure for this study. For that I would recommend putting this figure at the very last one as a conclusion. In the abstract, the authors stressed the function of CPSF30-L. Is it possible to put CPSF30-L also in the summary model?

*Response: Thanks for the kind suggestion. We have swapped the figure order and put CPSF30-L in the re-drawed summary model (Fig. 8 in the revised manuscript).*

Minor question:

1. In figure legend of Figure3, the 'a' and 'b' were put in wrong place. As the figure shows, 'a' represents ect2-1 compared to WT and 'b' represents ect2/3/4 compared to WT.

*Response: Sorry for the mistake, and we have corrected it.*

**Second round of review**

**Reviewer 1**

The authors have performed substantial experiments to address my concerns. I am happy to recommend its publication.

**Reviewer 2**

The authors have addressed all the questions raised in my previous comments. Also, error in fig 3 labeling has been corrected.

I recommend the authors to go throught the main text to edit english language and grammar to improve clarity for readers.

**Authors Response**

**Point-by-point responses to the reviewers’ comments:**

Reviewer #2: The authors have addressed all the questions raised in my previous comments. Also, error in fig 3 labeling has been corrected.

I recommend the authors to go throught the main text to edit english language and grammar to improve clarity for readers.

*Response: Thank you so much for your professional comments. The manuscript has been thoroughly polished. Now the contexts have been presented in a better manner.*
